# Development and validation of a multidisciplinary educational booklet for kidney transplant recipients

**DOI:** 10.1590/0034-7167-2024-0103

**Published:** 2025-03-14

**Authors:** Juliane Custodio de Andrade, Andressa Santos Ferreira Brito, Kelly Aparecida Vieria dos Santos, Ariane Polidoro Dini, Angélica Gonçalves Silva Belasco, Renata Cristina Gasparino

**Affiliations:** I Universidade Estadual de Campinas. Campinas, São Paulo, Brazil; II Hospital Irmãos Penteado. Campinas, São Paulo, Brazil; III Universidade Federal de São Paulo. São Paulo, São Paulo, Brazil

**Keywords:** Validation Studies, Health Education, Biomedical Technology, Patient Safety, Kidney Transplant, Estudios de Validación, Educación para la Salud, Tecnología Biomédica, Seguridad del Paciente, Trasplante de Riñón

## Abstract

**Objectives:**

to develop and validate the content of a multidisciplinary booklet for patients who have undergone kidney transplantation.

**Methods:**

this methodological study was conducted in five stages: 1) literature review; 2) identification and organization of domains; 3) content validation; 4) formatting of the booklet; and 5) evaluation by the target audience. Content validity was analyzed using the Content Validity Index (CVI) and modified Kappa. The Content Validity Ratio (CVR) was also calculated to assess relevance.

**Results:**

the booklet was validated after four rounds of evaluation by a panel of experts, achieving a global average of CVI = 0.98, modified Kappa = 0.97, and CVR = 0.77. The target audience evaluation reached a 99.7% agreement rate.

**Conclusions:**

the developed booklet demonstrated evidence of content validity, being clear, engaging, and informative, making it suitable for use by both professionals and the target audience.

## RESUMEN

**Objetivos:**

construir y validar un folleto multidisciplinario para pacientes sometidos a trasplante renal.

**Métodos:**

estudio metodológico en cinco etapas: 1) revisión bibliográfica; 2) organización de dominios e inserción de figuras; 3) validación del contenido; 4) formateo del folleto; y 5) evaluación por el público objetivo. La validez de contenido se analizó con el Índice de Validez de Contenido (IVC) y el Kappa modificado. Se calculó la Razón de Validez de Contenido (RVC) para medir la relevancia.

**Resultados:**

el folleto fue validado tras cuatro rondas de evaluación por expertos, con un IVC de 0,98, Kappa de 0,97 y RVC de 0,77. La evaluación del público objetivo mostró un 99,7% de concordancia.

**Conclusiones:**

el folleto mostró validez de contenido, siendo claro, interesante y completo, apto para uso por profesionales y pacientes.

## INTRODUCTION

The first kidney transplant (TX) took place in Boston in 1954^(1)^. In Brazil, kidney transplants began in 1964, and since then, the number of procedures has steadily increased^(2)^. Various complications can occur after TX, especially organ rejection and the development of infections, which are most prevalent in the first year post-transplant^(3)^, as this is the period of highest immunosuppressant use^(4)^.

To minimize or even prevent these events, it is essential to prioritize patient education with the goal of encouraging behavioral changes^(5)^ aimed at addressing the risk factors they are exposed to^(6)^.

The health education process has evolved significantly with scientific and technological advances, leading to the development of various educational technologies (ETs)^(7-9)^. The development of ETs should not be limited to materials and equipment but must be theoretically grounded, creative, innovative, engaging, and accessible in order to maximize the achievement of goals according to the needs of the target audience^(10)^.

For the construction of educational materials, a solid theoretical foundation is required. As part of this process, it is essential to utilize scientific evidence^(11)^ and validate the material with specialists in the field to ensure a thorough analysis of all content^(12)^. Subsequently, it is crucial to have the material evaluated by the target audience^(13)^.

There is a notable scarcity of multidisciplinary educational materials in the field of kidney transplantation, and studies focused on the construction and validation of such materials have significant potential for technical-scientific, social, and innovative impact. Therefore, it is believed that developing a booklet, considered an ET, will be beneficial for both professionals and patients. From a professional standpoint, the booklet can provide standardized guidance on the essential care that patients need to implement post-TX. For patients and caregivers, this tool will provide quick access to information based on the literature and validated by experts, assisting in resolving doubts, preventing complications, and ultimately contributing to patient safety.

## OBJECTIVES

To develop and validate the content of a booklet with guidance from a multidisciplinary team for patients who have undergone kidney transplants.

## METHODS

### Ethical considerations

The research was conducted in accordance with Resolution 466/12 of the National Health Council. The project was approved by the Research Ethics Committee of the institution responsible for data collection and the university affiliated with the study. All participants signed the Informed Consent Form (ICF).

### Study design, period, and location

This methodological study was developed in five stages: literature review; identification and organization of domains and items related to the guidance patients need from nursing, medical, pharmacy, physiotherapy, and nutrition teams, as well as the inclusion of illustrative figures; content validation; booklet formatting; and evaluation of the booklet by the target audience^(13)^.

The study was conducted from October 2020 to February 2022 at a public university hospital in the interior of São Paulo State.

### Data analysis and statistics

To evaluate the percentage of agreement among the experts, the Content Validity Index (CVI)^(13)^ and modified Kappa^(14)^ were calculated. CVI and modified Kappa values higher than 0.90 and 0.74, respectively, were considered satisfactory^(13,14)^. For relevance evaluation, the Content Validity Ratio (CVR) was used, with the minimum acceptable ratio set at 0.78 or higher^(15)^. The evaluation of the booklet by the target audience was performed using a percentage agreement calculation.

### Sample and inclusion/exclusion criteria

The inclusion criteria for the expert sample were professionals with at least one year of specialization or clinical experience in nephrology. Experts who did not respond to the form within the stipulated timeframe were excluded. For the target audience sample, 15 patients hospitalized who had undergone kidney transplantation at least four days prior and were clinically stable postoperatively were included, as well as 15 patients who had undergone kidney transplantation one month prior and were in outpatient follow-up. In both groups, the inclusion criteria were: first-time kidney TX recipients, over 18 years old, and literate.

### Study Protocol

In the first stage, a literature review was conducted, covering the past five years, on the care that kidney TX patients should receive after hospital discharge. To guide data collection, the following question was formulated: *What are the main guidelines that kidney transplant patients need to receive to prevent complications?* Subsequently, the following descriptors were defined and used: Validation studies, Health education, Health technology, Kidney TX, across the following databases and platforms: National Library of Medicine and National Institute of Health (PubMed), Latin American and Caribbean Health Sciences Literature (LILACS), Medical Literature Analysis and Retrieval System Online (MEDLINE), Scientific Electronic Library Online (Scielo), and Cumulative Index to Nursing & Allied Health Literature (CINAHL).

After selecting the relevant articles, abstracts were read, and a critical analysis of the included studies was performed. Additionally, information from protocols made available by the Ministry of Health (MS), the National Health Surveillance Agency (ANVISA), and the World Health Organization (WHO) was considered. At this point, a conceptual structure was established for developing the multidisciplinary educational content for the booklet^(12,13)^.

In the second stage, the domains and guidance that patients need to receive from nursing, medical, pharmacy, physiotherapy, and nutrition teams were identified. Subsequently, free and copyright-free illustrative images were selected to make the booklet more engaging, accessible, and easy to understand.

In the third stage, with the goal of evaluating content validity, ten professionals from the multidisciplinary team (nurses, pharmacists, physiotherapists, nutritionists, and physicians) involved in the care of kidney TX patients at the institution where the study was conducted were invited, by convenience sampling, to participate. Those who accepted were given access to an online form, built on the Google Forms^®^ platform, with the first screen containing the ICF, followed by a questionnaire to assess the personal and professional profile of the participant.

Participants were then asked to assess the clarity and representativeness of the content. For this purpose, each guideline was accompanied by a five-point Likert scale: 0) I cannot assess, as the item is outside my area of expertise; 1) Unclear and unrepresentative item; 2) The item needs significant revision to be clear and representative; 3) The item needs minor revision to be clear and representative; 4) The item is clear and representative.

For relevance evaluation, the experts used a four-point Likert scale: 0) I cannot assess, as the item is outside my area of expertise; 1) Unnecessary item; 2) Useful but unnecessary item; 3) Essential item.

Responses marked as zero were not considered in the calculations for the CVI, modified Kappa, and CVR^(15)^. For the items that did not meet the established test thresholds, the experts’ suggestions were incorporated for a second round of evaluation^(12,13)^. After content validation by the experts, the pre-final version of the booklet was formatted to be evaluated by the target audience. The final stage, referred to as the pre-test, involved the evaluation of the booklet by the target audience.

Hospitalized patients received a printed copy of the booklet and kept it for 24 to 48 hours. Meanwhile, outpatient care patients kept the booklet until their next follow-up appointment (two to seven days). After this period, patients were asked to complete a characterization form containing personal and clinical data and a questionnaire with questions related to the evaluation of the organization/appearance, writing style, and comprehension, as well as the motivation provided by the booklet^(16)^.

## RESULTS

In the first stage of the study, the literature review resulted in 25 articles, two theses, and three protocols, all of which were critically analyzed. From this review, in the second stage, 128 pieces of information were developed and conceptually distributed across five domains: 1) General guidelines and care after TX; 2) Vaccination recommendations; 3) Physical activity recommendations; 4) Medication usage; 5) Nutritional recommendations, in addition to useful contact information and references.

In the third stage, for the content validation process, of the ten professionals invited, eight agreed to participate, six of whom (75.00%) were female, and three (37.50%) had experience in validation studies. The average age was 35.38 years (±6.63), and the average time of practice in nephrology was 5.29 years (±5.52). Table 1 presents the remaining characterization variables of the expert sample.

**Table 1:** Characteristics of the experts (n=8), Campinas, São Paulo, Brazil, 2021

Variable	n	%
Education		
Nurse	2	25.00
Pharmacist	2	25.00
Physiotherapist	2	25.00
Nutritionist	1	12.50
Physician	1	12.50
Level of education		
Bachelor's degree	1	12.50
Lato sensu postgraduate in nephrology	2	25.00
Stricto sensu postgraduate	5	62.50
Time working in or having worked in nephrology		
<1 year	1	12.50
2 to 5 years	4	50.00
6 to 10 years	2	25.00
>11 years	1	12.50
Current workplace		
Other Wards	3	37.50
Nephrology Ward	2	25.00
Nephrology Outpatient Clinic	1	12.50
Other	1	25.00
Current position		
Clinical	2	25.00
Managerial	2	25.00
Teaching/Education	2	25.00
Clinical/Teaching/Education	2	25.00

The booklet was sent to the specialists, and after evaluation, the results related to the clarity, representativeness, and relevance of each item were obtained (Table 2).

**Table 2:** Evaluation of the clarity, representativeness, and relevance of the items in the first round of evaluation by the specialists (n=8), Campinas, São Paulo, Brazil, 2021

Items	CVI^*^	KAPPA	CVR ¨	Conduct	Items	CVI^*^	KAPPA	CVR ¨	Conduct
**1**	1.00	1.00	1.00	Maintained	**65**	0.83	0.82	0.33	Excluded
**2**	1.00	1.00	0.75	Maintained	**66**	1.00	1.00	0.67	Maintained
**3**	1.00	1.00	1.00	Maintained	**67**	0.83	0.82	0.33	Maintain/Exclude
**4**	1.00	1.00	0.75	Maintained	**68**	0.83	0.82	0.33	Excluded
**5**	1.00	1.00	0.67	Maintained	**69**	1.00	1.00	1.00	Maintained
**6**	1.00	1.00	1.00	Maintained	**70**	1.00	1.00	0.67	Excluded
**7**	1.00	1.00	0.75	Maintained	**71**	1.00	1.00	0.67	Excluded
**8**	1.00	1.00	1.00	Maintained	**72**	0.83	0.82	0.33	Excluded
**9**	0.86	0.85	0.75	Modified	**73**	1.00	1.00	0.67	Maintained
**10**	1.00	1.00	1.00	Maintained	**74**	1.00	1.00	0.43	Excluded
**11**	1.00	1.00	1.00	Maintained	**75**	1.00	1.00	0.43	Excluded
**12**	0.88	0.87	1.00	Modified	**76**	1.00	1.00	0.43	Maintained
**13**	1.00	1.00	1.00	Maintained	**77**	0.86	0.85	0.71	Excluded
**14**	1.00	1.00	1.00	Maintained	**78**	0.86	0.85	0.71	Excluded
**15**	0.88	0.87	1.00	Modified	**79**	0.86	0.85	0.71	Excluded
**16**	1.00	1.00	1.00	Maintained	**80**	0.86	0.85	0.71	Excluded
**17**	1.00	1.00	0.50	Maintained	**81**	0.86	0.85	0.71	Excluded
**18**	1.00	1.00	1.00	Maintained	**82**	0.86	0.85	0.71	Excluded
**19**	0.88	0.87	0.25	Excluded	**83**	0.86	0.85	0.71	Excluded
**20**	1.00	1.00	1.00	Maintained	**84**	0.86	0.85	0.71	Excluded
**21**	1.00	1.00	1.00	Maintained	**85**	0.86	0.85	0.71	Excluded
**22**	1.00	1.00	1.00	Maintained	**86**	0.86	0.85	0.71	Excluded
**23**	1.00	1.00	0.50	Maintained	**87**	0.86	0.85	0.71	Excluded
**24**	1.00	1.00	0.50	Maintained	**88**	0.86	0.85	0.71	Excluded
**25**	1.00	1.00	1.00	Maintained	**89**	0.86	0.85	0.71	Excluded
**26**	1.00	1.00	1.00	Maintained	**90**	0.86	0.85	0.71	Excluded
**27**	1.00	1.00	1.00	Maintained	**91**	0.86	0.85	0.71	Excluded
**28**	0.88	0.87	1.00	Modified	**92**	0.86	0.85	0.71	Excluded
**29**	0.88	0.87	1.00	Modified	**93**	0.86	0.85	0.71	Excluded
**30**	0.83	0.82	0.43	Excluded	**94**	1.00	1.00	1.00	Maintained
**32**	1.00	1.00	0.71	Maintain/Exclude	**95**	1.00	1.00	1.00	Maintained
**33**	0.83	0.82	0.43	Excluded	**96**	1.00	1.00	1.00	Maintained
**34**	1.00	1.00	0.43	Excluded	**97**	1.00	1.00	1.00	Maintained
**35**	0.83	0.82	0.43	Excluded	**98**	1.00	1.00	0.71	Maintained
**36**	0.83	0.82	0.43	Excluded	**99**	1.00	1.00	0.43	Maintained
**37**	0.83	0.82	0.71	Excluded	**100**	1.00	1.00	1.00	Maintained
**38**	1.00	1.00	0.43	Excluded	**101**	1.00	1.00	1.00	Maintained
**39**	0.83	0.82	0.43	Excluded	**102**	1.00	1.00	1.00	Maintained
**40**	0.83	0.82	0.43	Excluded	**103**	1.00	1.00	0.75	Maintained
**41**	1.00	1.00	1.00	Maintained	**104**	0.80	0.76	1.00	Modified
**42**	1.00	1.00	1.00	Maintained	**105**	0.80	0.76	0.60	Maintain/Exclude
**43**	1.00	1.00	1.00	Maintained	**106**	0.80	0.76	1.00	Maintained
**44**	1.00	1.00	0.75	Maintained	**107**	0.80	0.76	0.60	Excluded
**45**	1.00	1.00	0.50	Maintained	**108**	1.00	1.00	0.60	Maintained
**46**	1.00	1.00	0.75	Maintained	**109**	0.80	0.76	1.00	Modified
**47**	1.00	1.00	1.00	Maintained	**110**	1.00	1.00	1.00	Maintained
**48**	1.00	1.00	1.00	Maintained	**111**	0.75	0.67	-0.50	Excluded
**49**	1.00	1.00	1.00	Maintained	**112**	0.60	0.42	-0.20	Excluded
**50**	1.00	1.00	1.00	Maintained	**113**	0.60	0.42	-0.50	Excluded
**51**	1.00	1.00	0.67	Maintained	**114**	0.80	0.76	-0.50	Excluded
**52**	0.83	0.82	0.67	Modified	**115**	1.00	1.00	1.00	Maintained
**53**	1.00	1.00	0.67	Maintained	**116**	0.80	0.76	-0.50	Excluded
**54**	1.00	1.00	0.67	Maintained	**117**	0.60	0.42	-1.00	Excluded
**55**	1.00	1.00	0.67	Maintained	**118**	0.80	0.76	0.20	Modified
**56**	0.83	0.82	0.33	Modified	**119**	1.00	1.00	1.00	Maintained
**57**	1.00	1.00	1.00	Maintained	**120**	0.80	0.76	0.60	Maintain/Exclude
**58**	1.00	1.00	1.00	Maintained	**121**	0.80	0.76	0.60	Excluded
**59**	1.00	1.00	0.67	Maintained	**122**	0.83	0.82	0.67	Excluded
**60**	1.00	1.00	0.67	Maintained	**123**	0.83	0.82	0.67	Excluded
**61**	0.83	0.82	0.33	Modified	**124**	0.83	0.82	0.67	Excluded
**62**	1.00	1.00	0.67	Excluded	**125**	0.83	0.82	0.67	Modified
**63**	1.00	1.00	0.67	Maintained	**126**	0.83	0.82	1.00	Maintain/Exclude
**64**	0.83	0.82	0.33	Modified	**127**	1.00	1.00	1.00	Maintained
					**128**	1.00	1.00	1.00	Maintained

*Notes: CVI - Content Validity Index; ¨CVR - Content Validity Ratio.*

After the first evaluation, 38 items achieved scores of 1.00 in IVC, Modified Kappa, and CVR, and were therefore retained without any modifications. Item 106 was maintained despite having CVI and Modified Kappa values below the reference, with a CVR of 1.00, as it received no suggestions for alteration and was considered relevant by all experts. Another 24 items, although presenting an CVR below 0.78, did not receive a score of 1 (unnecessary item) from any expert during the relevance assessment. Since no suggestions for modifications were made and the items achieved CVI and Modified Kappa values of 1.00, they were also retained without changes.

Among the total items, seven achieved CVI and Modified Kappa scores of 1.00, indicating that they were clear and represented the domain, but had unsatisfactory CVR values and were excluded because they received a score of 1 (unnecessary item) from at least one expert in the relevant area. Another 39 items were excluded due to unsatisfactory CVI, Modified Kappa, or CVR values, with no suggestions for changes, and because they received a score of 1 (unnecessary item) from at least one expert in the relevant area.

Five items had unsatisfactory CVI, Modified Kappa, or CVR values but did not receive any score of 1 (unnecessary item) or suggestions for changes. Therefore, these items were returned for a second round of evaluation, where the experts were asked whether the item should be retained or excluded.

For another 13 items that achieved CVI values below the recommended level but received modification suggestions, it was decided to alter them and submit them for re-evaluation. In addition to the 128 initial items, the experts suggested adding 25 more. These items, along with the 18 that had unsatisfactory results in the first round, were sent for a second round of evaluation.

In this second round, seven experts participated, as one physiotherapist did not respond to the questionnaire within the established timeframe. In this round, three items were excluded, and three others did not achieve satisfactory CVI and Modified Kappa values but received suggestions for alteration.

Regarding relevance assessment, another 13 items achieved CVR scores below the recommended threshold. However, none received a score of 1 (unnecessary item) or suggestions for changes, and thus were retained as they were, given that the CVI and Modified Kappa achieved maximum values.

In the third round, the same experts from the second round participated, but a consensus could still not be reached. Therefore, an online meeting was scheduled with the committee members. In the fourth round, only three specialists (a physician, a nurse, and a nutritionist) participated, as they were the experts on the topics related to the respective items. During the meeting, the items were discussed, modified, and, with the consensus of all, retained with the proposed changes.

At the end of this stage, the global average of clarity and representativeness evaluation was CVI = 0.98 and Modified Kappa = 0.97. Regarding the relevance of the retained items, the CVR was 0.77.

After this stage, the pre-final version of the multidisciplinary guidance booklet for kidney TX patients was composed of 111 items. After formatting, organization, and adjustments, 30 patients who had undergone kidney transplantation evaluated the educational booklet, with 99.7% agreeing with the organization, writing style, appearance, and motivation provided by the booklet.

## DISCUSSION

The present study developed and validated the content of a booklet to assist the multidisciplinary team in guiding patients who have undergone kidney TX, facilitating quick and easy access to the necessary information for both patients and their caregivers in the post-TX period. Strengthening health education in post-TX care is considered an essential factor for the success of the procedure, as it contributes to reducing complications and hospital readmissions^(17)^. To ensure effective knowledge transmission, the use of ETs is increasingly being explored. The literature highlights the use of videos, text messages, printed documents^(9)^, apps, websites, and booklets^(10)^. It is worth noting that, despite technological advancements, the availability of printed booklets is still necessary, especially for patients with limited access to mobile devices and the internet.

The booklet, due to its accessibility to various audiences, has proven to be an excellent tool for healthcare professionals in the educational process. These materials have gained traction as they provide information within various health contexts^(18-21)^.

The initial version of the booklet was developed with 128 items, distributed across five domains. The construction phase was based on scientific evidence obtained through a literature review^(10,21-23)^. After the article search, the material was read and critically analyzed, with the most relevant information extracted to meet the study’s objectives.

The content validation phase is crucial for allowing the critical analysis of the items that will comprise the booklet, conducted by specialists to ensure that the material contains necessary and up-to-date information related to the proposed objective^(24)^.

The content validation process was carried out by a multidisciplinary team (nurses, pharmacists, physiotherapists, a nutritionist, and a physician), covering various fields of knowledge. These professionals, as seen in other studies, had experience in the subject matter and varying levels of education, with most having postgraduate degrees (stricto sensu)^(22,25-27)^. The formation of a multidisciplinary and experienced committee has been increasingly used^(20,26,27)^ in content validation, adding significant contributions and higher quality to the topics covered.

Eight specialists participated in the study, a number consistent with various content validation studies that used a minimum of five professionals^(20-22,26,27)^. Although the literature varies in its recommendations regarding the number of specialists^(25,28)^, the present study followed the guidelines of national authors^(13)^.

In the present study, four rounds of evaluation were necessary. It is worth noting that several studies reached a consensus among specialists after just one evaluation round^(25-27,29)^. The need for four rounds may reflect the expertise in the subject matter and the high level of training and commitment of the specialists involved in developing the material. Another possible explanation for the four rounds is that this study differed from others by using more than one calculation to assess content validity, whereas many studies with high levels of agreement among specialists used only the CVI calculation^(20,22,25,29)^.

The use of the three-point scale CVR also made the evaluation more rigorous, which aligns with another study that used the same calculation^(23)^. Research employing a four-point scale for CVR evaluation showed a higher percentage of agreement from the first round^(30)^.

At the end of the content validation phase, the pre-final version of the booklet consisted of 111 items, divided into four domains: 1) Guidelines and general care after TX, focusing on the prevention and identification of complications; 2) Recommendations and contraindications related to physical activities during the post-TX period; 3) Medication use, with an emphasis on immunosuppressants; and 4) Nutritional recommendations (healthy eating, food preparation care, and water intake), as well as useful phone numbers and references.

These domains align with the needs reported by patients in a previous study, in which the difficulties identified were related to performing daily tasks, medication routines, diet, and exercise^(31)^.

In the final stage of the study, the evaluation of the booklet by the target audience showed a high level of agreement among participants, allowing the conclusion that the developed booklet is appropriate for its intended audience. The patients who participated in this stage evaluated the booklet in terms of organization, writing style, appearance, and motivation for reading, similar to other studies^(18,31,32)^. However, it is important to note that other research did not include this fundamental stage of evaluating the clarity, interest, and motivation that the material evokes in the target audience^(20,21,23,28)^.

The inclusion of images related to the topics covered made the booklet more attractive, sparking the reader’s interest. For the booklet developed in the present study, royalty-free images were sourced from the internet and were considered of good quality and effective in capturing the readers’ attention, which differs from other studies that used a specific design for educational or communication materials^(18,19,25)^.

The constructed and validated booklet is available^(33)^ for use, and new studies can be designed to test the effectiveness of the educational process using this material, patient empowerment, and its impact on reducing complications.

### Study limitations

The committee was composed of specialists from the study location and selected by convenience, which may reflect the specific characteristics of the institution where the research was conducted. However, it is important to note that most of the topics addressed reflect the main studies on post-TX care. Additionally, the absence of a specific design in the development and/or evaluation of the material can also be considered a limitation in the development of the technology.

### Contributions to the field

It is believed that the use of this ET in clinical practice and teaching will contribute to the standardization of evidence-based information, covering the most relevant topics in post-TX care. The availability of this material is expected to facilitate access to information in a quick, clear, and objective manner, empowering patients in their self-care and ensuring their safety.

## CONCLUSIONS

The booklet was developed based on the literature and demonstrated evidence of content validity, being clear, engaging, and containing necessary information for the target audience.

## Data Availability

https://doi.org/10.20396/ISBN9786587100326 PDF
