# Supplementary material for: Development and validation of a multidisciplinary educational booklet for kidney transplant recipients
Source: Rev Bras Enferm. 2025 Mar 14;78(1):e20240103. doi: 10.1590/0034-7167-2024-0103 (PMC11913046; doi:10.1590/0034-7167-2024-0103)
Supplement: Supplementary file 1 [file 0034-7167-reben-78-01-e20240103-suppl01.pdf]

Hospital de Clínicas
UNICAMP

FACULDADE DE ENFERMAGEM

Cartilha Educativa Multiprofissional para Transplantados Renais

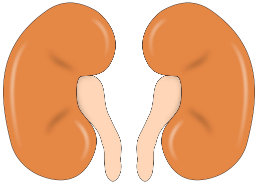

<u>Autor</u>: Juliane Custodio de Andrade e Andressa Santos Ferreira Brito

<u>Ilustrador</u>: Andressa Santos Ferreira Brito

<u>Revisores:</u>

Alessandra Martins Lother

Carolina Ferreira Cantelle

Juliana Furlan Ravagnani

Lara Paro Dias

Leonardo Figueiredo Camargo

Marcelo Gustavo Pereira

Patrícia Tamy Shinta

Thayssa de Moraes Oliveira

<u>Editor:</u> Renata Cristina Gasparino

Campinas - SP

UnicampBFCM

2023

UNICAMP
FACULDADE DE CIÊNCIAS MÉDICAS
BIBLIOTECA

Ficha catalográfica elaborada por
Maristella Soares dos Santos
CRB-8/8402

An24c Andrade, Juliane Custodio de, 1986-

Cartilha educativa multiprofissional para transplantados renais [recurso eletrônico] / Juliane Custodio de Andrade, Andressa Santos Ferreira Brito ; revisores Alessandra Martins Lother... [et al.] ; editor Renata Cristina Gasparino. - 2. Ed. - Campinas, SP : Edição do autor, 2023.
79 p. : il. ; PDF

ISBN 978-65-87100-32-6
Modo de acesso World Wide Web:
https://www.bibliotecadigital.unicamp.br/bd/index.php/detalhes-material/?code=114212

1. Transplante de rim. 2. Transplantados. 3. Equipe de assistência ao paciente. 4. Educação de pacientes. II. Brito, Andressa Santos Ferreira, 1994-. III. Lother, Alessandra Martins, 1972-. IV. Gasparino, Renata Cristina, 1981-. V. Título.

CDD 617.4610592

Entendendo a cartilha..

Essa cartilha foi desenvolvida e validada por profissionais capacitados (médicos, enfermeiros, nutricionistas, fisioterapeutas e farmacêuticos). E é composta por 4 temas principais:

1) Orientações e cuidados gerais após o transplante;
2) Recomendações sobre atividade física;
3) Uso de medicações e
4) Recomendações nutricionais.

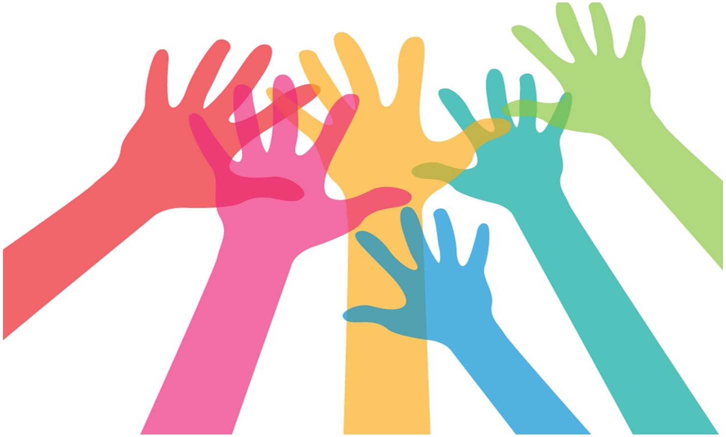

Parabéns! Você ganhou um novo rim!

Estamos muito contentes por você. E para evitar complicações, elaboramos essa cartilha explicativa com o resumo de tudo que você precisa saber para aproveitá-la ao máximo! Vamos lá:

Os primeiros meses após o transplante necessitam de muita atenção, pois é nesse período que ocorrem as principais complicações. É muito importante seguir as recomendações para prevenir complicações como: rejeição e infecção e também melhorar sua qualidade de vida.

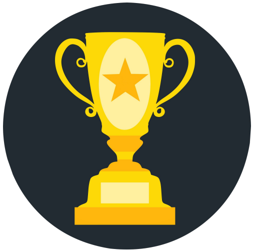

Parabéns! Você ganhou um novo rim!

Mas afinal o que são os rins?

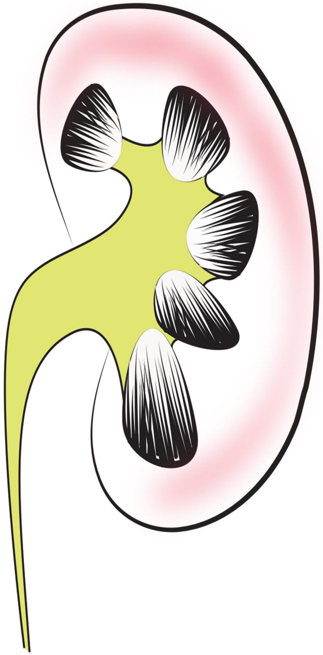

Entre outras coisas, eles são responsáveis por filtrar o sangue e eliminar substâncias nocivas ao organismo, como amônia, uréia e ácido úrico.

Eles também secretam hormônios que são importantes para o bom funcionamento do nosso corpo.

Guyton, 2011

Orientações gerais após a alta hospitalar

Você receberá um caderno do transplantado!

Leve sempre com você o Caderno do Transplantado, nele estarão contidas informações sobre seu transplante e as medicações que fará uso! Se necessário, ao final da cartilha poderá ser realizado anotação da sua pressão arterial, quantidade de líquido que está bebendo e quantidade de urina que está eliminando!

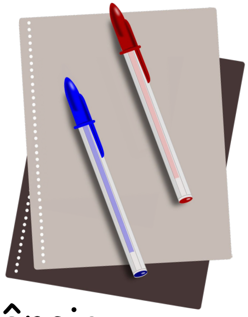

As anotações que realizar no caderno serão vistas pelo médico no dia da consulta no ambulatório e por outros profissionais de saúde que estão envolvidos na sua assistência.

Orientações gerais após a alta hospitalar

Alta hospitalar:

Os pontos da sua cirurgia poderão ser retirados a partir de 7 dias de acordo com a avaliação médica antes da alta hospitalar ou no dia da consulta ambulatorial.

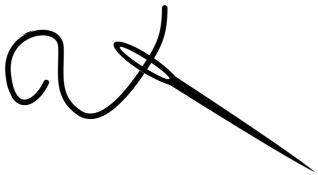

Attias et al; 2019

Risco de infecção

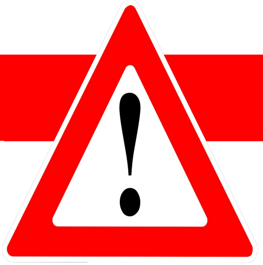

O risco de infecções pós transplante é dividido em três períodos:

- Até quatro semanas após o transplante: o maior risco são as infecções causadas pelo procedimento cirúrgico, como por exemplo na ferida operatória e no trato urinário.
- De quatro semanas até seis meses: os riscos são das infecções oportunistas como a tuberculose e citomegalovírus.
- A partir de seis meses: O risco é semelhante a população geral, porém, é importante lembrar que as infecções nos pacientes transplantados são mais graves.

Jackson KR at al 2021, Attias P at al 2019, Tizo MJ at al 2015

Infecções oportunistas

As infecções mais prevalentes são:

1º Trato urinário

2º Infecções sistêmicas

3º Ferida operatória

4º Trato respiratório

5º Mucosa orofaríngea

6º Gastrointestinal

7º Renal

8º Genital

Jackson KR at al 2021, Attias P at al 2019, Kang at al 2018, Lucena et al 2013

Atenção aos sinais de alerta

- Febre
- Mal estar
- Diarréia
- Inchaço
- Dor ou Dificuldade ao urinar
- Urina com sangue
- Tosse ou falta de ar
- Inchaço e dor no local da cirurgia

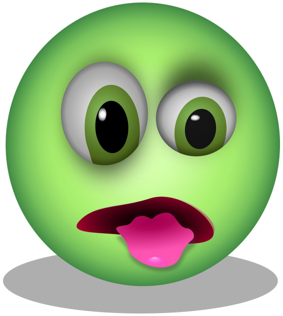

Hassanein 2020, Natori Y 2018, Kang 2018, Pires et al 2015

Risco de rejeição

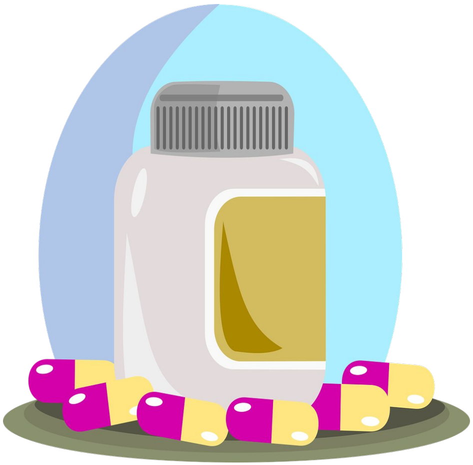

- É um dos maiores problemas pós transplante renal, as células de defesa do organismo podem reconhecer o novo rim como um corpo estranho e atacá-lo. Para evitar que isso ocorra é extremamente necessário o uso dos imunossupressores, no início as doses são maiores e serão diminuídas gradativamente.

Baker at al 2017, Tizo MJ at al, 2015

Tipos de rejeição

- <u>Rejeição hiperaguda:</u> Pode acontecer nas primeiras 24 horas após o transplante.

- <u>Rejeição aguda:</u> pode ocorrer entre o 3° dia até 3 meses após o transplante, é o mais comum e neste caso, existe um tratamento efetivo.

- <u>Rejeição crônica:</u> pode ocorrer ao longo da evolução do transplante, levando a uma perda lenta e progressiva da função do rim transplantado.

Tizo MJ et al; 2015

Sinais de alerta de Rejeição

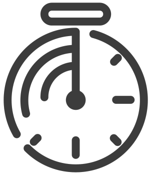

Pressão alta.

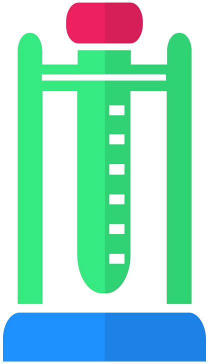

Exames laboratoriais alterados, exemplo: creatinina aumentada.

Diminuição da quantidade de urina;
Urina espumosa (pode indicar proteína na urina).

Tantisattamo E et al; 2020, Diena et al; 2019, Baker et al; 2017

Cuidados Gerais

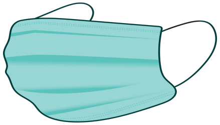

Use máscara, dentro e fora de casa. Se a máscara for de pano a mesma deverá ser lavada diariamente, se optar por máscara descartável a mesma deverá ser trocada a cada 6 horas ou se sujidade ou umidade.

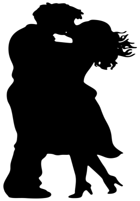

A relação sexual é permitida após liberação médica. Converse com a equipe sobre sua saúde sexual! Proteja-se, use camisinha. O exame de papanicolau deve ser feito regularmente!

Evite engravidar no primeiro ano pós transplante. Sua gestação deverá ser programada para evitar a perda do novo rim.

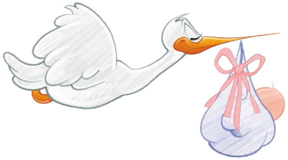

Tavares MS et al; 2020, Mariano et al; 2019, Baker at al; 2017

Cuidados Gerais

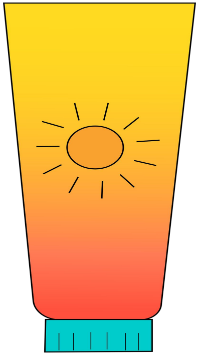

Não se esqueça: use filtro solar sempre!

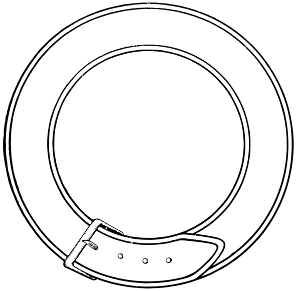

Use a cinta elástica diariamente nos três primeiros meses após o transplante.

Manickavasagar et al; 2020, Kaszak DB et al; 2020

Cuidados Gerais

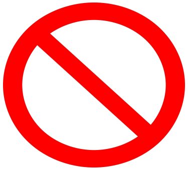

Não fume, não use drogas e evite ingerir bebidas alcoólicas.
Se precisar, solicite ajuda!

Mantenha a casa sempre limpa e arejada, troque a roupa de cama 1x por semana.

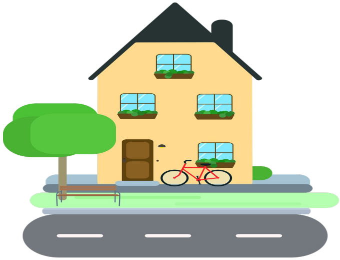

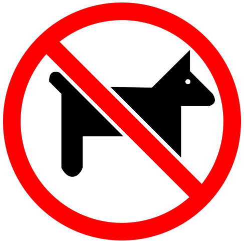

Evite contato com animais. Nós sabemos como eles são importantes, mas neste momento, sua imunidade está baixa e, por isso, eles podem lhe transmitir doenças.

Manickavasagar et al; 2020, Kaszak DB et al; 2020

Previna infecção!

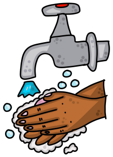

Lave as mãos: antes das refeições, após voltar da rua, antes e após usar o banheiro.

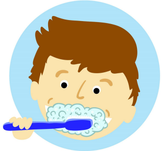

Escove os dentes após as refeições com uma escova macia e não se esqueça do fio dental. Vá ao dentista regularmente.

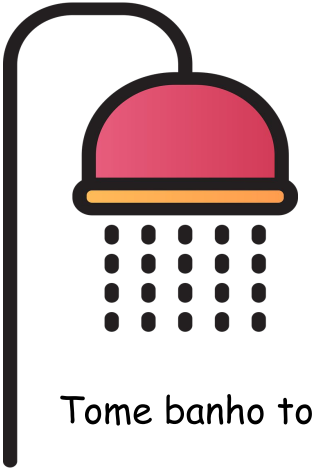

Não se esqueça de realizar higiene íntima SEMPRE que urinar ou evacuar. Não use calcinha/cueca molhada e mantenha a parte íntima seca.

Tome banho todos os dias.

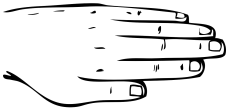

Mantenha as unhas sempre curtas e limpas, e tenha sempre muito cuidado para não colocar a mão da boca.

Velioglu A et al; 2021, Hillier MD; 2020, Schönfeld B et al; 2019, Sihra N; 2018

Previna infecção

Evite locais fechados e com aglomeração de pessoas, principalmente pessoas doentes. Evite receber visitas quando chegar em casa.

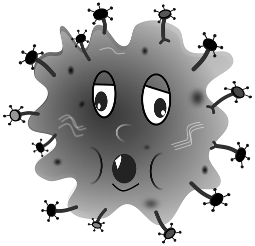

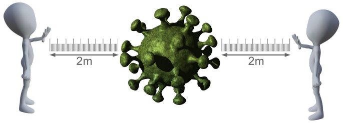

Caso alguém venha te visitar, permita um por vez mantendo uma distância segura (dois metros) e uma boa circulação do ar.

Lupi D et al; 2020

Voltando a rotina...

Assim que possível, você poderá voltar ao trabalho, se esse for o seu desejo após liberação médica. O retorno ao trabalho é realizado pelo médico da empresa onde você trabalha e não pelo médico do transplante. O médico do transplante irá fornecer um relatório que será entregue ao médico da empresa

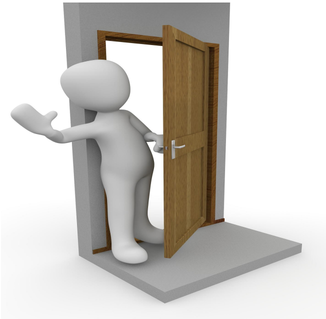

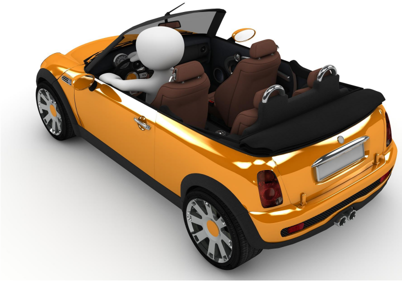

Você só poderá dirigir, após liberação médica. Não esqueça de ser cuidadoso no trânsito e usar o cinto de segurança

Vacinação

Leve sempre sua carteira de vacinação nas consultas do ambulatório. Consulte a equipe de saúde sempre quando for realizar uma nova vacina e não tome nenhuma vacina sem liberação médica.

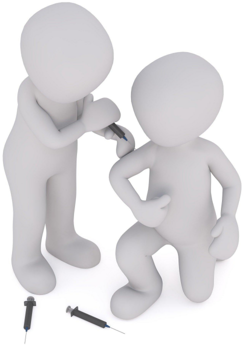

ABTO; 2018, Baker et al; 2017

Atividade Física

A cicatrização total ocorre de seis a oito semanas após o transplante, evite: levantar ou empurrar objetos pesados, realizar atividades que produzam ou aumentem a dor no local da cirurgia. Exercícios de alta intensidade, somente após liberação médica.

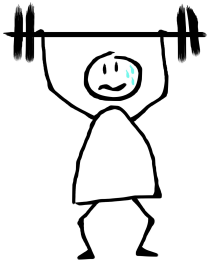

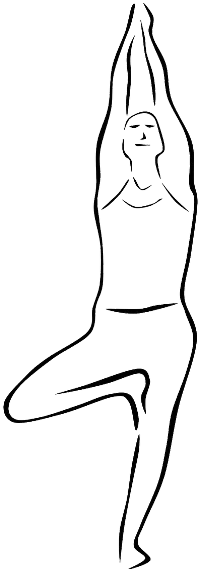

Caminhadas leves são permitidas mas caso apresente falta de ar ou cansaço, suspenda e comunique seu médico no dia da consulta! Os exercícios físicos ajudam na recuperação da força física, porém, é importante a orientação de um profissional sobre o programa de exercícios mais recomendados para você.

Zhu Q et al; 2021, Hu Y et al; 2019, Takahashi A et al; 2018

Atividade Física

Os exercícios físicos podem ser intensificados aos poucos, mas EVITE as seguintes atividades:

- ❑ Esportes violentos e/ou traumáticos, que possam provocar choque na região abdominal (futebol, handebol, jiu-jitsu, karatê).
- ❑ Exercícios físicos durante episódios de rejeição.
- ❑ Atividades sexuais com posições ou situações que provoquem dor.

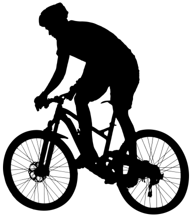

Andar de bicicleta e nadar em piscinas limpas são excelentes exercícios, assim que houver liberação médica.

É importante suspender qualquer atividade física se houver falta de ar excessiva.

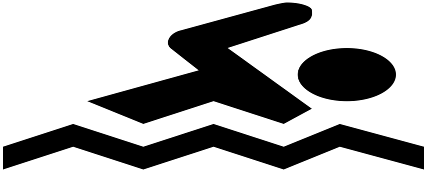

Wilkinson TJ et al; 2020, Takahashi A et al; 2018, Greenwood SA et al; 2015

Atividade Física

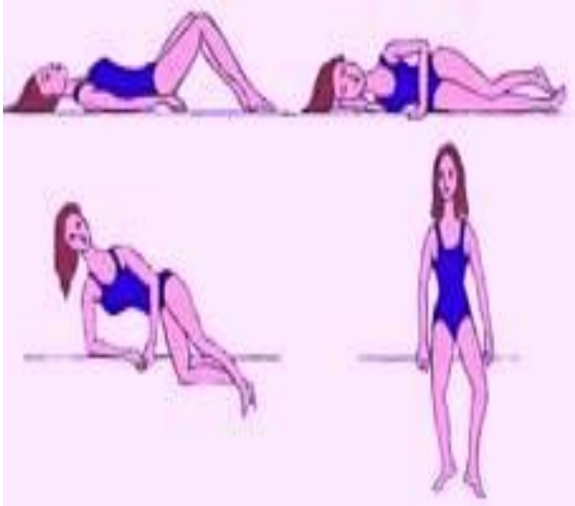

Nos primeiros meses deve-se evitar grandes esforços físicos abdominais, como por exemplo, levantar-se e deitar-se na cama bruscamente.

Deve-se virar para o lado contrário ao operado, flexionar um pouco os joelhos e inclinar o corpo para sair da cama, deixando as pernas caírem para fora da mesma.

Atividade Física

Segue algumas dicas de atividades físicas leves que podem ser feitas em casa com liberação médica:

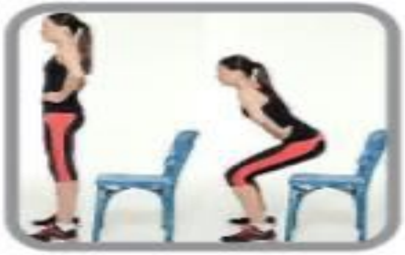

Sentar e levantar da cadeira sem ajuda das mãos.

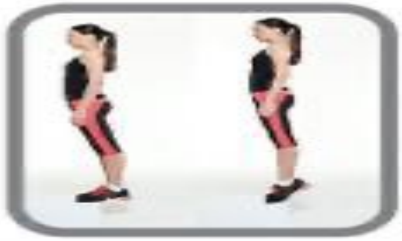

Elevação do calcanhar;

Greenwood SA et al; 2015

Atividade Física

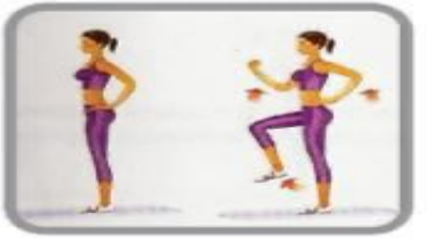

Marcha estacionária (andar sem sair do lugar)

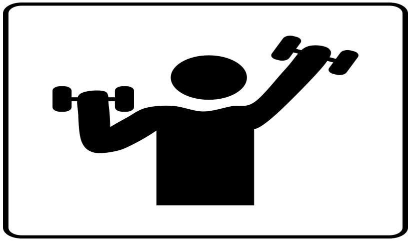

Elevação de braços com ou sem peso leve

Caminhada em algum espaço livre da casa como corredor, quintal ou cômodo com mais espaço.

Greenwood SA et al; 2015

Uso das medicações

Para prevenir a rejeição do seu novo rim é necessário o uso de medicamentos imunossupressores. Esses medicamentos deixam a sua imunidade baixa, aumentando o risco de infecções.

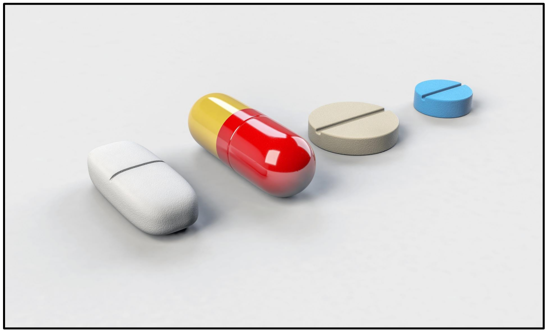

Mas seu uso é muito importante para o sucesso do transplante!

Baker at al; 2017

Uso das medicações

- Confira e anote a quantidade de medicamentos em casa antes de ir à consulta médica e comunique a equipe se estiver acabando ou a quantidade de medicamentos não for suficiente até a próxima consulta. Se necessário o médico fará nova receita para você retirar os medicamentos na farmácia.
- Só use medicações liberadas pela equipe do transplante
- Em caso de vômito logo após ingerir os comprimidos, não tome uma dose extra. Aguarde o próximo horário

MS; 2016

Uso das medicações

- Caso esqueça de tomar o remédio, tome os comprimidos assim que se lembrar. Não duplique a dose para compensar uma dose esquecida. Caso o horário tenha atrasado muito, como mais de 6 horas, pule a dose esquecida e aguarde o horário normal.
- Os comprimidos devem ser engolidos inteiros. Nunca quebrados, amassados ou cortados. Não mastigue-os e tome sempre com água.
- Manter a medicação na embalagem original, em local fresco e protegido da luz solar. Evitar locais úmidos, como banheiro.

MS; 2016

Uso das medicações

- Mantenha todos os medicamentos fora do alcance de crianças e animais de estimação.
- Algumas medicações podem apresentar efeitos colaterais.
- Você poderá sentir alguns sintomas, mas **não** deve suspender a medicação por conta própria.
- Avise a equipe de saúde o quanto antes, para que possamos estudar a melhor forma de te ajudar.

MS; 2016

Uso das medicações

Lembre-se:

<u>Você não está sozinho!</u>

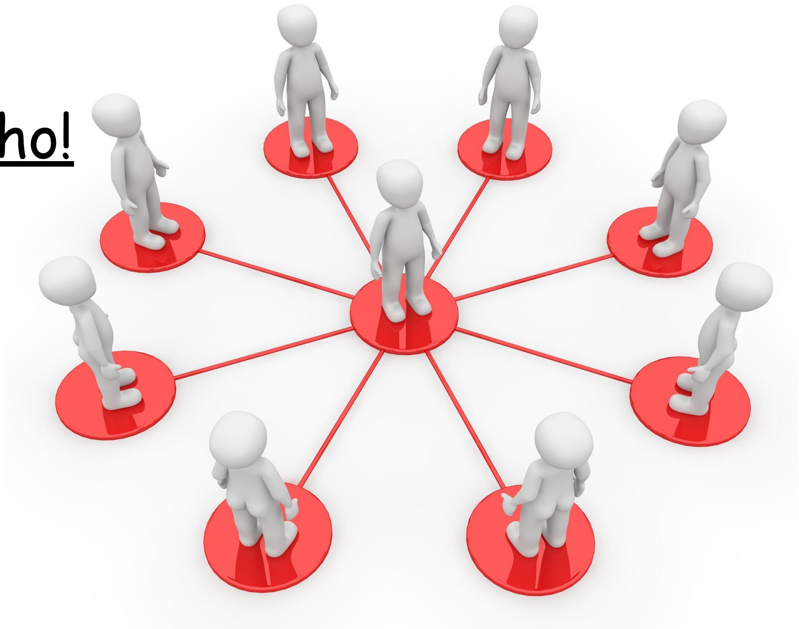

Medicações: Tacrolimo

Tacrolimo (imunossupressor): é indicado para prevenir rejeição.

*Os comprimidos devem ser ingeridos com água.
*Permanecer 1 hora em jejum antes e 1 hora em jejum depois de tomar o comprimido.Nesse período, se você quiser, poderá beber água.
*O médico irá solicitar exames com frequência para verificar a concentração de Tacrolimo no sangue e ajustar a dose.

Comunique a equipe de saúde se apresentar:

- Diarréia (mais de 5 evacuações líquidas por dia) por mais de um dia;
- Dor abdominal acompanhada ou não de outros sintomas (calafrios, febre, náuseas ou vômito)
- Nos dias de exame de sangue, não tome a medicação antes do exame, porém traga com você e tome após a coleta de sangue.

Bulário eletrônico- MS/ Anvisa/ EMS; 2020, Baker et al; 2017

Medicações: Micofenolato

<u>Micofenolato (imunossupressor):</u> é indicado para prevenir rejeição.

*Tome esse medicamento sempre com água. Informe seu médico se você estiver tomando outros medicamentos como: antiácidos que contenham magnésio e alumínio (omeprazol, lansoprazol, pantoprazol), antibióticos, norfloxacino e metronidazol.

*Este medicamento reduz o efeito do anticoncepcional oral.

*Não tome comprimidos quebrados.

<u>Comunique a equipe de saúde se apresentar:</u>

- Diarréia, manchas roxas sem causa aparente ou sangramentos.

Bulário eletrônico- MS/ Anvisa/ EMS; 2020, Baker et al; 2017

Medicações: Sirolimo (Rapamune)

Sirolimo (imunossupressor): é indicado para prevenir rejeição.

* Serão realizados exames periodicamente para ajuste de doses, conforme necessidade.

Informe ao seu médico se você ou alguém de sua família tem câncer de pele e se você tem ou teve problemas no fígado ou alguma doença que possa ter afetado este órgão. Essa informação é importante para a prescrição da dose correta.

Bulário eletrônico- MS/ Anvisa/ EMS; 2020, Baker et al; 2017

Medicações: Azatioprina

<u>Azatioprina (imunossupressor):</u> é indicado para prevenir rejeição.

<u>Informe seu médico ou farmacêutico</u>:

*Se você sofre de doença no fígado;

*Se estiver tomando os seguintes medicamentos: captopril, cimetidina, alopurinol, oxipurinol, tiopurinol, furosemida e anticoagulantes.

Bulário eletrônico- MS/Anvisa; 2019

Medicações: Ciclosporina

Ciclosporina: é indicado para prevenir rejeição.

*Não use ciclosporina com toranja (grapefruit) ou suco de toranja, pois isto pode alterar o efeito de ciclosporina

Bulário eletrônico- MS/ Anvisa/ Fund. Remédio Popular; 2019

Medicações: Prednisona

Prednisona: é indicado para prevenir rejeição.

*O comprimido deve ser tomado com um pouco de líquido, pela manhã;

*Esse medicamento pode aumentar seu apetite.

*Faça exercícios moderados para prevenir obesidade e osteoporose.

Bulário eletrônico- MS/Anvisa/ Aché farmacêuticos; 2020

Medicações: Bactrim

Bactrim (sulfametoxazol + trimetoprima): Antibiótico, utilizado para prevenir infecção.

*Os comprimidos de Bactrim® devem ser administrados por via oral, de preferência após uma refeição e com quantidade suficiente de líquido.

Bulário eletrônico- MS/Anvisa/ Farmoquímica; 2020

Medicações: Ácido fólico

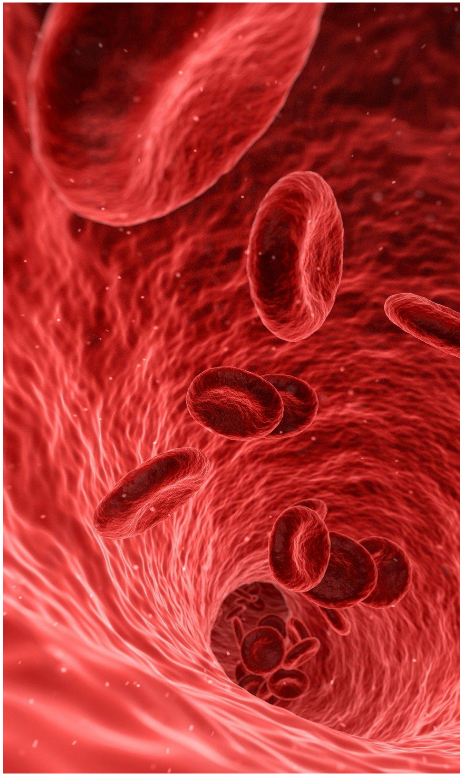

<u>Ácido fólico (vitamina B9):</u> é um suplemento nutricional.

Ele ajuda na produção de diversas substâncias que formam as proteínas presentes no corpo humano, é uma vitamina essencial para o funcionamento do organismo.

Bulário eletrônico- MS/ Anvisa/ Funed;2016

Medicações: Vitamina B

<u>Complexo B:</u> Você utilizará essa medicação para prevenir deficiência de vitaminas.

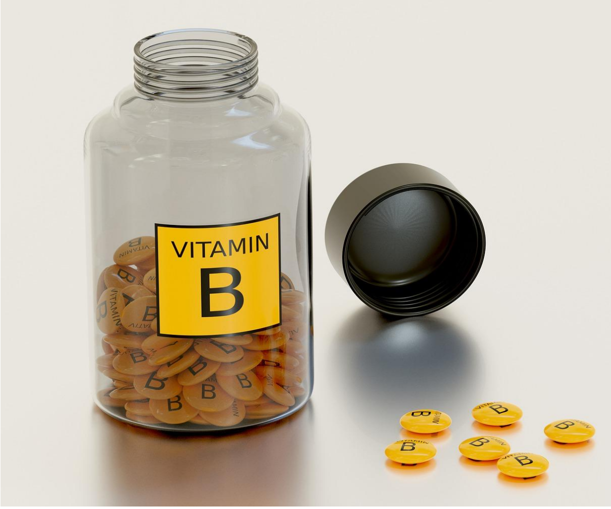

* Este medicamento deve ser utilizado por via oral, não deve ser partido, aberto ou mastigado.

Bulário eletrônico- MS/Anvisa/ EMS; 2020

Uso das medicações

Não use medicamentos indicados por outras pessoas que não sejam os profissionais de saúde envolvidos na sua assistência.

Existem sintomas que são parecidos em diferentes doenças. O uso de remédios sem recomendação de um profissional de saúde pode prejudicar você!

<u>Algumas dicas podem ajudá-lo:</u>

- Antes de iniciar o tratamento com o medicamento, leia a receita médica com atenção.
- Nunca mude a dose diária recomendada!

Uso das medicações

- Faça o tratamento completo, não pare o uso porque teve melhora dos sintomas. Os medicamentos precisam ser tomados por todo o tratamento indicado, principalmente durante o uso de antibióticos e outros medicamentos de uso controlado.

- Caso apresente dificuldade em organizar a rotina de utilização diária de seus medicamentos, solicite ajuda do farmacêutico ou da equipe que esta cuidando de você. Vamos encontrar uma maneira para te auxiliar nesta organização.

Uso das medicações

- Observe os seus sintomas e se você tem sentido alguma alteração após o início do uso da medicação (coceiras, tontura, dores de cabeça, alteração de humor, etc.). Informe o profissional de saúde que o prescreveu.
- Evite a ingestão de bebidas alcóolicas. Medicamentos e álcool são uma combinação que deve ser evitada.
- Mantenha seus medicamentos protegidos. De preferência em sua própria embalagem e com a bula, longe do alcance de crianças e animais domésticos, para evitar acidentes.

ANS, 2020

Nutrição pós Transplante Renal

A rotina da alimentação no pós transplante renal deve prover uma ingestão adequada de nutrientes para diminuir o risco de infecção, ajudar na cicatrização da ferida operatória e na manutenção da massa muscular. Esses cuidados trazem um melhor funcionamento do novo rim. Além disso, uma alimentação adequada é essencial pós transplante renal, pois evita/controla hipertensão, diabetes, aumento do colesterol e triglicérides sanguíneo.

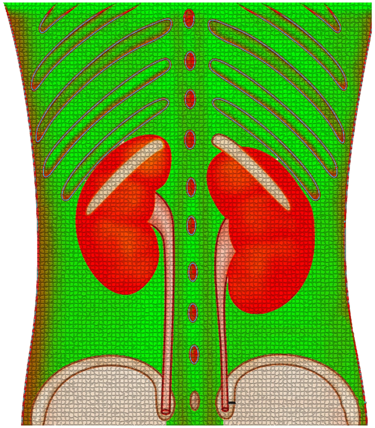

Hortegal, Dias; 2015, Baker et al; 2017

Nutrição pós Transplante Renal: Proteínas

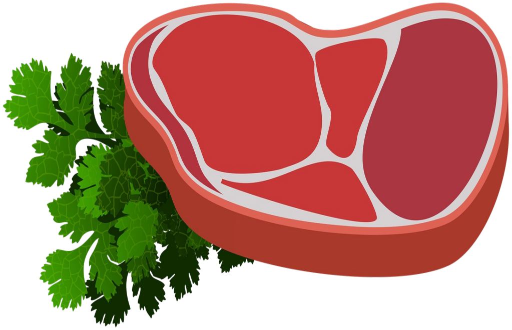

As proteínas são muito importantes para o organismo. São fontes de proteínas: Alimentos de origem animal, tais como carne de todos os tipos, leite e derivados e ovos.

MS; 2014, Hortegal, Dias; 2015

Nutrição pós Transplante Renal: Sódio

O sal de cozinha (cloreto de sódio) e outros compostos químicos que contêm sódio são muito usados na produção de alimentos industrializados. Seu consumo deve ser mínimo para diminuir o risco de doenças do coração e ajudar no controle da pressão arterial.

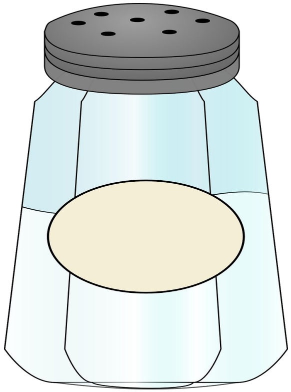

Use ervas ou temperos e não sal, para contribuir com o sabor dos alimentos. Fique longe dos temperos prontos que contêm alta concentração de sal. Sugerimos que use sachês de 1g por prato S (a quantidade equivalente a uma tampinha de caneta BIC® por dia)

MS; 2014, Hortegal, Dias; 2015

Nutrição pós Transplante Renal: Sódio

TEMPEROS: UMA SOLUÇÃO PARA POUCO SAL.

Os temperos que indicamos a seguir têm ótima qualidade para conferir um toque especial às preparações do dia-a-dia e são ótimas opções para contornar a diminuição de sal: aipo, alho, alho poró, cebola, louro, páprica, pimentões, tomate, salsinha.

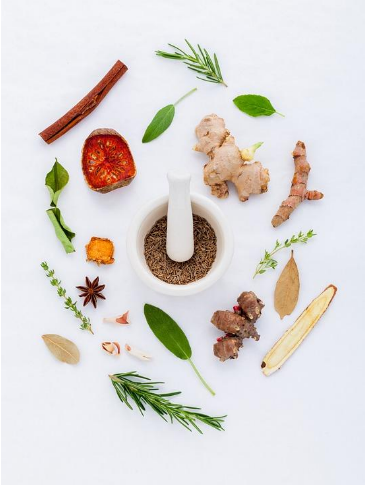

MS; 2014, Hortegal, Dias; 2015

Nutrição pós Transplante Renal: Sódio

Dicas de onde usar temperos:

- Alecrim: batata e frango;
- Gengibre: frutas e peixes;
- Hortelã: saladas e grelhados;
- Manjericão: saladas e massas;
- Noz-moscada: verduras, carnes e massas;
- Orégano: pratos que contém tomate na preparação;
- Pimenta: carnes, verduras e massas;
- Salsinha: saladas, sopas e molhos;
- Curry: frango, peixes, verduras e massas;
- Coentro, cebolinha, cheiro-verde: todas as preparações.

MS; 2014, Hortegal, Dias; 2015

Nutrição pós Transplante Renal: Recomendações

Sódio:

O sódio da alimentação deve ser limitado em até 3g/dia, inclusive para as pessoas que possuem a pressão arterial normal. Em caso de pressão alta ou retenção de líquido, o valor é mais restrito, entre 1 a 2 g/dia.

Lípidos:

São alimentos energéticos assim como os carboidratos. Porém, devem ser consumidos em quantidade moderada, para que não ocorram complicações como as dislipidemias. recomenda-se utilizar óleos em pequenas quantidades para cozinhar e usar azeite de oliva para saladas. Não usar banha para cozinhar, evitar frituras e o consumo de maionese, creme de leite, toucinho, bacon, entre outros.

Hortegal, Dias; 2015

Nutrição pós Transplante Renal: Recomendações

A alimentação deve ser fracionada de 3 em 3 horas e divida em 6 refeições por dia:

café da manhã

lanche da manhã

almoço

lanche da tarde

jantar

ceia

Uma dieta equilibrada é composta por todos os grupos de alimentos: carboidratos, proteínas, lipídios (gorduras). Sugere-se acrescentar à dieta alimentos ricos em fibras como os cereais integrais, aveia, frutas,. verduras e legumes.

Hortegal, Dias; 2015

Nutrição pós Transplante Renal: Recomendações

As vitaminas e minerais devem estar presentes na nossa alimentação todos os dias. Orienta-se consumir pelo menos:

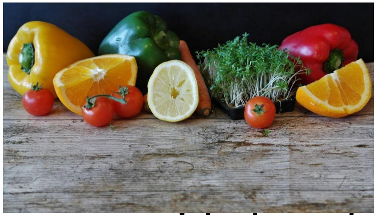

3 porções de frutas

2 porções de legumes

2 porções de salada crua por dia para que as necessidades de vitaminas e minerais sejam alcançadas.

Variedade: o hábito de consumir vários tipos de alimentos ajuda a promover a introdução de diferentes nutrientes.

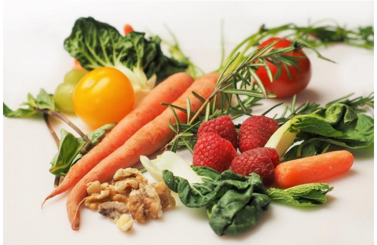

Hortegal, Dias; 2015

Cuidado com os alimentos

Procedimentos para Seleção, Lavagem e Desinfecção de Frutas, Legumes e Verduras:

Na hora da compra, não selecionar o alimento que contém:

1. Casca ou polpa que estejam mole, manchada, mofada ou de cor diferente do habitual;

2. Folhas, talos ou raízes murchas, mofadas ou deterioradas;

3. Qualquer mudança na cor, na consistência ou no cheiro característico.

Cuidado com os alimentos

Na hora do preparo:

- ❑ Retirar as folhas e partes deterioradas;
- ❑ Lavar em água corrente os vegetais folhosos, folha a folha, e as frutas e legumes, um a um;
- ❑ Colocar de molho, por 10 minutos, em água tratada, utilizando produto adequado para esse fim (diluir 1 colher de sopa de água sanitária própria para alimentos ou pingar 10 gotas de hipoclorito de sódio à 10% para 1 litro de água, ou conforme recomendação do fabricante);
- ❑ Enxaguar em água corrente;
- ❑ Cortar os alimentos para a montagem dos pratos com as mãos e objetos bem lavados;
- ❑ Manter na geladeira até a hora de servir;

Cuidado com os alimentos

- (gema mole, gemada ou preparações que os ovos não são totalmente cozidos, como mousses, por exemplo), leite e seus derivados não pasteurizados.

- Não consuma alimentos preparados fora de casa por no mínimo 3 meses após o transplante. Se necessário, leve consigo alimentos não perecíveis ou armazenados em bolsa térmica

MS; 2014

Água

- Após o transplante, você deverá beber uma grande quantidade de água, que será informada pelo médico no momento de sua alta;

- Nos primeiros 3 meses você poderá utilizar água mineral engarrafada de até 5 litros ou água fervida.

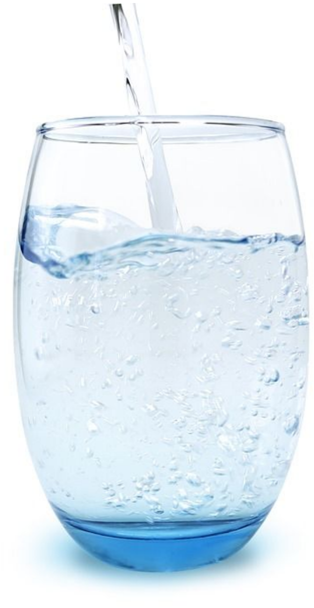

MS; 2014

Água

- Após 3 meses do transplante você poderá utilizar água de purificadores ou filtros de parede, certificados pelo INMETRO ou galão de água mineral.

- Observe sempre a validade do filtro e troque-o segundo as especificações do fabricante. Em caso de não ser possível a compra de galões de água e não tiver filtros em casa, a alternativa é ferver a água da torneira por no mínimo 5 minutos, começando a contar este tempo após a presença de borbulhas.

- ATENÇÃO: os mesmos cuidados para preparo de gelos ou sucos.

MS; 2014

Telefones úteis

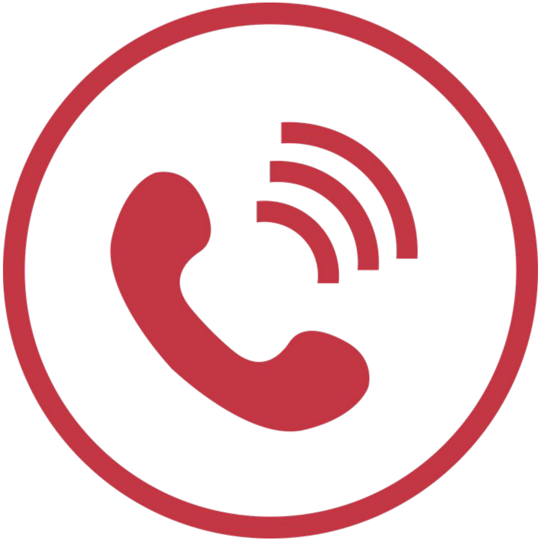

AMBULATÓRIO
(19) 35217881
Seg á Sexta
das 8h às 17h

ENFERMARIA
(19) 35217359
24 horas por dia

Referências

Attias P, Melica G, Boutboul D, De Castro N, Audard V, Stehlé T, et al. Epidemiology, Risk Factors, and Outcomes of Opportunistic Infections after Kidney Allograft Transplantation in the Era of Modern Immunosuppression: A Monocentric Cohort Study. J Clin Med. 2019;8(5):594.

Baker RJ, Mark PB, Patel RK, Stevens KK, Palmer N. Renal association clinical practice guideline in post-operative care in the kidney transplant recipient. BMC Nephrol [Internet]. 2017;18(1):174.

Brasil. Ministério da Saúde. Secretaria de Atenção à Saúde. Departamento de Atenção Hospitalar e de Urgência. Segurança do paciente no domicílio / Ministério da Saúde, Secretaria de Atenção à Saúde, Departamento de Atenção Hospitalar e de Urgência. – Brasília : Ministério da Saúde, 2016.

Bulário eletrônico – Ministério da Saúde/Anvisa/Fundação Ezequiel Dias – FUNED – 2016. Ácido fólico. Disponível em: <http://www.anvisa.gov.br/datavisa/fila_bula/frmResultado.asp>. [Acesso em 30 de Novembro de 2020].

Referências

Bulário eletrônico - Ministério da Saúde/ Anvisa/ Fund. para o remédio popular 2019. Azatioprina. Disponível em: <http://www.anvisa.gov.br/datavisa/fila_bula/frmResultado.asp#>.[Acesso em 18 de Dezembro de 2020].

Bulário eletrônico- Ministério da Saúde/ Anvisa/ FARMOQUÍMICA S/A -2020. Bactrim. Disponível em: <http://www.anvisa.gov.br/datavisa/fila_bula/frmResultado.asp#> [Acesso em 30 de Novembro de 2020].

Bulário eletrônico - Ministério da Saúde/ Anvisa/ EMS S/A 2020 Complexo B. Disponível em:<http://www.anvisa.gov.br/datavisa/fila_bula/frmResultado.asp#>[Acesso em 30 de Novembro de 2020].

Bulário eletrônico- Ministério da Saúde/ Anvisa/aché laboratórios farmacêuticos s.a 2020. Prednisona. Disponível em: <http://www.anvisa.gov.br/datavisa/fila_bula/frmResultado.asp#>. [Acesso em 30 de novembro de 2020].

Referências

Bulário eletrônico - Ministério da Saúde/ Anvisa/ wyeth indústria farmacêutica ltda ems 2020. Rapamune. Disponível em: <http://www.anvisa.gov.br/datavisa/fila_bula/frmResultado.asp#> [Acesso em 30 de Novembro de 2020]

Bulário eletrônico - Ministério da Saúde/ Anvisa/accord farmacêutica ltda2020. Micofenolato de mofetila. Disponível em: <http://www.anvisa.gov.br/datavisa/fila_bula/frmResultado.asp#> [Acesso em Novembro de 2020].

Diena D, Messina M, De Biase C, Fop F, Scardino E, Rossetti MM, et al. Relationship between early proteinuria and long term outcome of kidney transplanted patients from different decades of donor age. BMC Nephrol. 2019;20(1):1–15.

Greenwood SA, Koufaki P, Mercer TH, Rush R, O'Connor E, Tuffnell R, at al. Aerobic or Resistance Training and Pulse Wave Velocity in Kidney Transplant Recipients: A 12-Week Pilot Randomized Controlled Trial (the Exercise in Renal Transplant. Am J Kidney Dis. 2015;66(4):689-98.

Referências

Guyton AC, Hall JE. Tratado de Fisiologia Médica. 12. ed. Rio de Janeiro: Elsevier, 2011.

Hillier MD. Using effective hand hygiene practice to prevent and control infection. Nurs Stand. 2020;35(5):45-50.

Ministério da Saúde. Guia alimentar para a população brasileira., secretaria de atenção à saúde, departamento de atenção Básica. – 2. ed. – Brasília; 2014.

Hortegal EV, Dias RSCD. Nutrição para a prevenção da doença renal crônica. Universidade Federal do Maranhão. UNA-SUS/UFMA. - São Luís, 2015.

Hu Y, McArthur A, Yu Z. Early postoperative mobilization in patients undergoing abdominal surgery: a best practice implementation project. JBI Database System Rev Implement Rep. 2019;17(12):2591-2611.

Jackson KR, Motter JD, Bae S, Kernodle A, Long JJ, Werbel W, et al. Characterizing the landscape and impact of infections following kidney transplantation. Am J Transplant. 2021;21(1):198–207.

Referências

Jehn U, Schütte-Nütgen K, Bautz J, Pavenstädt H, Suwelack B, Thölking G, Heinzow H, Reuter S. Cytomegalovirus Viremia after Living and Deceased Donation in Kidney Transplantation. J Clin Med. 2020;9(1):252.

Kang CI, Kim J, Park DW, et al. Clinical Practice Guidelines for the Antibiotic Treatment of Community-Acquired Urinary Tract Infections. Infect Chemother. 2018;50(1):67-100.

Kaszak DB, Biegańska MJ, Dąbrowska I. Occurrence of various pathogenic and opportunistic fungi in skin diseases of domestic animals: a retrospective study. BMC Vet Res. 2020;16(1):248.

Lucena AF, Echer IC, Assis MCS, Ferreira SAL, Teixeira CC, Steinmetz QL. Complicações infecciosas no transplante renal e suas implicações às intervenções de enfermagem: revisão integrativa. Rev enferm UFPE. 2013;7(esp):953-959.

Referências

Lupi D, Binda B, Montali F, Natili A, Lancione L, Chiappori D, Parzanese I, Maccarone D, Pisani F. Transplant Patients' Isolation and Social Distancing Because of COVID-19: Analysis of the Resilient Capacities of the Transplant in the Management of the Coronavirus Emergency. Transplant Proc. 2020;52(9):2626-2630.

Manickavasagar R, Thuraisingham R. Post renal-transplant malignancy surveillance. Clin Med J R Coll Physicians London. 2020;20(2):142–5.

Mariano S, Guida JPS, Sousa MV, Parpinelli MA, Surita FG, Mazzali M, et al. Pregnancy among women with kidney transplantation: A 20-Years Single-Center Registry. Rev Bras Ginecol e Obstet. 2019;41(7):419–24.

Natori Y, Albahrani S, Alabdulla M, Vu J, Chow E, Husain S, et al. Risk factors for surgical site infection after kidney and pancreas transplantation. Infect Control Hosp Epidemiol. 2018;39(9):1042–8.

Referências

Pires BUA, Oliveira CM, Gouveia MRF, Oliveira JI. Pós - operatório de transplante Renal - Orientações para pacientes e familiares. Hospital das Clínicas de Porto Alegre. Rev educação em saúde, Vol 81. 2015.

Schönfeld B, Varga Á, Szakály P, Bán Á. Oral Health Status of Kidney Transplant Patients. Transplant Proc. 2019;51(4):1248-1250.

Sihra N, Goodman A, Zakri R, Sahai A, Malde S. Nonantibiotic prevention and management of recurrent urinary tract infection. Nat Rev Urol. 2018; 15: 750–776.

Sociedade Brasileira de Imunização. Guia de Imunização SBIM/ABTO -Transplante de Órgãos. 2019/2020. Disponível em: <https://sbim.org.br/publicacoes/guias/1128-guia-de-imunizacao-sbim-abto-transplante-de-orgaos-2019-2020>. [Acesso em 10 de Novembro de 2020].

Referências

Takahashi A, Hu SL, Bostom A. Physical Activity in Kidney Transplant Recipients: A Review. Am J Kidney Dis. 2018;72(3):433-443.

Tantisattamo E, Molnar MZ, Ho BT, et al. Approach and Management of Hypertension After Kidney Transplantation. Front Med (Lausanne). 2020;7:229.

Tavares MS at al. Recommendations Of The Brazilian Society Of Nephrology Regarding Pediatric Patients On Renal Replacement Therapy. 2020;32–5.

Tizo MJ, Macedo LC. Principais complicações e efeitos colaterais pós transplante renal. Rev Uningá Review. 2015;14(1):62-70.

Referências

Velioglu A, Guneri G, Arikan H, Asicioglu E, Tigen ET, Tanidir Y, Tinay İ, Yegen C, Tuglular S. Incidence and risk factors for urinary tract infections in the first year after renal transplantation. PLoS One. 2021;16(5):e0251036.

Wilkinson TJ, McAdams-DeMarco M, Bennett PN, Wilund K; Global Renal Exercise Network. Advances in exercise therapy in predialysis chronic kidney disease, hemodialysis, peritoneal dialysis, and kidney transplantation. Curr Opin Nephrol Hypertens. 2020;29(5):471-479.

Zhu Q, Yang J, Zhang Y, Ni X, Wang P. Early mobilization intervention for patient rehabilitation after renal transplantation. Am J Transl Res. 2021;13(6):7300-7305.

Referências Imagens

1. Universidade Estadual de Campinas [Logo] [Imagem da internet]. Campinas-SP. UNICAMP, Site Oficial, 1994 [Acesso em 17 de Agosto de 2021] 110 x 101 pixels. Disponível em: https://www.unicamp.br/unicamp/sites/default/files/inline-images/logo_124_0.png

2. Faculdade de Enfermagem da Universidade Estadual de Campinas [Logo] [Imagem da internet]. Campinas-SP. Fenf, Site Oficial. [Acesso em 17 de Agosto de 2021] 65 x 49 pixels. Disponível em: https://www.fenf.unicamp.br/sites/default/files/logo_grey_only.png

3. Rim Anatomia [Imagem da Internet] Pixabay. Banco de Imagens. [Acesso em 17 de Agosto de 2021] 960 x 680 pixels. Disponível em: https://pixabay.com/pt/vectors/rim-anatomia-humano-homem-%c3%b3rg%c3%a3o-147499/

4. Troféu [Imagem da Internet] Pixabay. Banco de Imagens. [Acesso em 17 de Agosto de 2021] 1600 x 1598 pixels. Disponível em: https://pixabay.com/es/vectors/victoria-premio-trofeo-taza-icono-5834110/

Referências Imagens

5. Rim [Imagem da Internet] Pixabay. Banco de Imagens. [Acesso em 17 de Agosto de 2021] 1061 x 1600 pixels. Disponível em: https://pixabay.com/ru/illustrations/%d0%bf%d0%be%d1%87%d0%ba%d0%b0-%d0%bf%d0%be%d1%87%d0%b5%d1%87%d0%bd%d1%8b%d0%b9-med-%d0%bc%d0%b5%d0%b4%d0%b8%d1%86%d0%b8%d0%bd%d1%81%d0%ba%d0%b8%d0%b9-1716229/

6. Rim [Imagem da Internet] Pixabay. Banco de Imagens. [Acesso em 17 de Agosto de 2021] 1061 x 1600 pixels. Disponível em: https://pixabay.com/ru/illustrations/%d0%bf%d0%be%d1%87%d0%ba%d0%b0-%d0%bf%d0%be%d1%87%d0%b5%d1%87%d0%bd%d1%8b%d0%b9-med-%d0%bc%d0%b5%d0%b4%d0%b8%d1%86%d0%b8%d0%bd%d1%81%d0%ba%d0%b8%d0%b9-1716229/

7. Rim [Imagem da Internet] Pixabay. Banco de Imagens. [Acesso em 17 de Agosto de 2021] 1061 x 1600 pixels. Disponível em: https://pixabay.com/ru/illustrations/%d0%bf%d0%be%d1%87%d0%ba%d0%b0-%d0%bf%d0%be%d1%87%d0%b5%d1%87%d0%bd%d1%8b%d0%b9-med-%d0%bc%d0%b5%d0%b4%d0%b8%d1%86%d0%b8%d0%bd%d1%81%d0%ba%d0%b8%d0%b9-1716229/

Referências Imagens

8. Grupo [Imagem da Internet] Pixabay. Banco de Imagens. [Acesso em 17 de Agosto de 2021] 1280 x 1280 pixels. Disponível em: https://pixabay.com/ru/illustrations/%d0%b3%d1%80%d1%83%d0%bf%d0%bf%d0%b0-%d1%82%d0%b5%d1%80%d0%b0%d0%bf%d0%b8%d1%8f-%d0%ba%d0%be%d0%bd%d1%81%d1%83%d0%bb%d1%8c%d1%82%d0%b8%d1%80%d0%be%d0%b2%d0%b0%d0%bd%d0%b8%d0%b5-2351896/

9. Material Escolar [Imagem da Internet] Pixabay. Banco de Imagens. [Acesso em 17 de Agosto de 2021] 978 x 1280 pixels. Disponível em: https://pixabay.com/pt/vectors/material-escolar-canetas-caderno-1977836/

10. Linha e agulha [Imagem da Internet] Pixabay. Banco de Imagens. [Acesso em 17 de Agosto de 2021] 1260 x 1280 pixels. Disponível em: https://pixabay.com/pt/vectors/agulha-de-costura-fio-reparar-312738/

11. Alerta [Imagem da Internet] Pixabay. Banco de Imagens. [Acesso em 17 de Agosto de 2021] 1280 x 1144 pixels. Disponível em: https://pixabay.com/pt/vectors/aten%c3%a7%c3%a3o-aviso-assinar-perigo-303861/

Referências Imagens

12. Atenção [Imagem da Internet] Pixabay. Banco de Imagens. [Acesso em 17 de Agosto de 2021] 1280 x 1144 pixels. Disponível em: https://pixabay.com/pt/vectors/aten%c3%a7%c3%a3o-aviso-assinar-perigo-303861/

13. Rosto doente. [Imagem da Internet] Pixabay. Banco de Imagens. [Acesso em 17 de Agosto de 2021] 1138 x 1280 pixels. Disponível em: https://pixabay.com/nl/vectors/grafisch-bah-smiley-bah-smiley-3943389/

14 Medicamento. [Imagem da Internet] Pixabay. Banco de Imagens. [Acesso em 17 de Agosto de 2021] 1280 x 934 pixels. Disponível em: https://pixabay.com/es/vectors/medicamento-cuenco-farmacia-salud-3270263/

15. Just in Time. [Imagem da Internet] Pixabay. Banco de Imagens. [Acesso em 17 de Agosto de 2021] 1920 x 1920 pixels. Disponível em: https://pixabay.com/ru/illustrations/just-in-time-jit-%d1%87%d0%b0%d1%81%d1%8b-%d0%b7%d0%bd%d0%b0%d1%87%d0%be%d0%ba-3750378/

Referências Imagens

16. Tubo de Ensaio. [Imagem da Internet] Pixabay. Banco de Imagens. [Acesso em 17 de Agosto de 2021] 1119 x 1280 pixels. Disponível em: https://pixabay.com/da/vectors/reagensglas-ikon-blod-handikap-5118698/

17. Urina. [Imagem da Internet] Pixabay. Banco de Imagens. [Acesso em 17 de Agosto de 2021] 1280 x 884 pixels. Disponível em: https://pixabay.com/pt/vectors/tarefa-amarelo-respingo-pintura-1528002/

18. Corona vírus. [Imagem da Internet] Pixabay. Banco de Imagens. [Acesso em 18 de Agosto de 2021] 1280 x 1020 pixels. Disponível em: https://pixabay.com/fi/vectors/naamio-korona-virus-covid-19-4982908/

19 Proibido. [Imagem da Internet] Pixabay. Banco de Imagens. [Acesso em 18 de Agosto de 2021] 1280 x 1280 pixels. Disponível em: https://pixabay.com/de/vectors/verboten-verbot-nein-tue-nicht-155564/

20. Cegonha. [Imagem da Internet] Pixabay. Banco de Imagens. [Acesso em 18 de Agosto de 2021] 1280 x 704 pixels. Disponível em: https://pixabay.com/pt/vectors/beb%c3%aa-p%c3%a1ssaro-entrega-f%c3%aamea-garota-1299514/

Referências Imagens

21. Casal. [Imagem da Internet] Pixabay. Banco de Imagens. [Acesso em 18 de Agosto de 2021] 1920 x 931 pixels. Disponível em: https://pixabay.com/pt/photos/casal-romance-amor-beijo-amantes-3064048/

22. Armação de cinto de fivela. [Imagem da Internet] Pixabay. Banco de Imagens. [Acesso em 18 de Agosto de 2021] 1272 x 1280 pixels. Disponível em:https://pixabay.com/ko/vectors/%EB%B2%84%ED%81%B4-%EB%B2%A8%ED%8A%B8-%EC%95%A1%EC%9E%90-1009320/

23. Casa de Campo. [Imagem da Internet] Pixabay. Banco de Imagens. [Acesso em 18 de Agosto de 2021] 1002 x 1280 pixels. Disponível em:https://pixabay.com/fr/vectors/maison-cottage-maison-d-habitation-2003069/

24. Proibido. [Imagem da Internet] Pixabay. Banco de Imagens. [Acesso em 18 de Agosto de 2021] 1280 x 1280 pixels. Disponível em: https://pixabay.com/de/vectors/verboten-verbot-nein-tue-nicht-155564/

Referências Imagens

25. Cegonha. [Imagem da Internet] Pixabay. Banco de Imagens. [Acesso em 18 de Agosto de 2021] 1280 x 704 pixels. Disponível em: https://pixabay.com/pt/vectors/beb%c3%aa-p%c3%a1ssaro-entrega-f%c3%aamea-garota-1299514/

26. Casal. [Imagem da Internet] Pixabay. Banco de Imagens. [Acesso em 18 de Agosto de 2021] 1920 x 931 pixels. Disponível em: https://pixabay.com/pt/photos/casal-romance-amor-beijo-amantes-3064048/

27. Armação de cinto de fivela. [Imagem da Internet] Pixabay. Banco de Imagens. [Acesso em 18 de Agosto de 2021] 1272 x 1280 pixels. Disponível em:https://pixabay.com/ko/vectors/%EB%B2%84%ED%81%B4-%EB%B2%A8%ED%8A%B8-%EC%95%A1%EC%9E%90-1009320/

28. Casa de Campo. [Imagem da Internet] Pixabay. Banco de Imagens. [Acesso em 18 de Agosto de 2021] 1002 x 1280 pixels. Disponível em:https://pixabay.com/fr/vectors/maison-cottage-maison-d-habitation-2003069/

Referências Imagens

29. Signo Animal. [Imagem da Internet] Pixabay. Banco de Imagens. [Acesso em 18 de Agosto de 2021] 1280 x 1280 pixels. Disponível em: https://pixabay.com/id/vectors/tanda-hewan-peliharaan-simbol-42533/

30. Protetor Solar. [Imagem da Internet] Pixabay. Banco de Imagens. [Acesso em 18 de Agosto de 2021] 805 x 1920 pixels. Disponível em: https://pixabay.com/pt/illustrations/protetor-solar-sol-per%c3%adodo-de-f%c3%a9rias-4969051/

31. Lavar as mãos. Imagem da Internet] Pixabay. Banco de Imagens. [Acesso em 18 de Agosto de 2021] 1920 x 1920 pixels. Disponível em: https://pixabay.com/es/illustrations/lavar-lavarse-las-manos-coronavirus-4958623/

32 Escovar os dentes. Imagem da Internet] Pixabay. Banco de Imagens. [Acesso em 18 de Agosto de 2021] 1280 x 1280 pixels. Disponível em: https://pixabay.com/zh/illustrations/brushing-teeth-tooth-dental-teeth-2351803

Referências Imagens

33. Banho d'agua. Imagem da Internet] Pixabay. Banco de Imagens. [Acesso em 18 de Agosto de 2021] 720 x 1280 pixels. Disponível em: https://pixabay.com/ru/vectors/%d0%b4%d1%83%d1%88-%d0%b2%d0%b0%d0%bd%d0%bd%d0%b0-%d0%b8%d0%ba%d0%be%d0%bd%d0%b0-%d0%b2%d0%b0%d0%bd%d0%bd%d0%b0%d1%8f-%d0%ba%d0%be%d0%bc%d0%bd%d0%b0%d1%82%d0%b0-5786673/

34. Mão e dedos. [Imagem da Internet] Pixabay. Banco de Imagens. [Acesso em 18 de Agosto de 2021] 1280 x 779 pixels. Disponível em: https://pixabay.com/zh/vectors/hand-fingers-body-human-skin-303168/

35. Vacina. [Imagem da Internet] Pixabay. Banco de Imagens. [Acesso em 18 de Agosto de 2021] 1280 x 1244 pixels. Disponível em: https://pixabay.com/pt/vectors/germe-doente-infec%c3%a7%c3%a3o-sa%c3%bade-41367/

36. Distanciamento social. [Imagem da Internet] Pixabay. Banco de Imagens. [Acesso em 18 de Agosto de 2021] 1920 x 1200 pixels. Disponível em: https://pixabay.com/zh/illustrations/social-distancing-virus-covid-19-4990640/

Referências Imagens

37. Porta entrada apartamento. [Imagem da Internet] Pixabay. Banco de Imagens. [Acesso em 18 de Agosto de 2021] 1920 x 1920 pixels. Disponível em:https://pixabay.com/pt/illustrations/porta-apartamento-entrada-bloqueado-1013696/

38. Mini conversível. [Imagem da Internet] Pixabay. Banco de Imagens. [Acesso em 18 de Agosto de 2021] 1920 x 1920 pixels. Disponível em: https://pixabay.com/no/illustrations/mini-konvertibel-sj%c3%a5f%c3%b8r-topp-ri-1027851/

39. Injeção. [Imagem da Internet] Pixabay. Banco de Imagens. [Acesso em 18 de Agosto de 2021] 1920 x 1920 pixels. Disponível em: https://pixabay.com/no/illustrations/mini-konvertibel-sj%c3%a5f%c3%b8r-topp-ri-1027851/

40. Levantamento de peso. [Imagem da Internet] Pixabay. Banco de Imagens. [Acesso em 18 de Agosto de 2021] 1920 x 1920 pixels. Disponível em: https://pixabay.com/pl/illustrations/podnoszenia-ci%c4%99%c5%bcar%c3%b3w-zm%c4%99czenie-1872377/

Referências Imagens

41. Ioga. Pose da árvore. [Imagem da Internet] Pixabay. Banco de Imagens. [Acesso em 18 de Agosto de 2021] 640 x 1280 pixels. Disponível em: https://pixabay.com/pt/vectors/ioga-pose-de-ioga-pose-de-%c3%a1rvore-32127/

42. Atleta Bicicleta. [Imagem da Internet] Pixabay. Banco de Imagens. [Acesso em 18 de Agosto de 2021] 1125 x 1280 pixels. Disponível em: https://pixabay.com/pt/vectors/atleta-bicicleta-ciclismo-humano-2025775/

43. Natação. [Imagem da Internet] Pixabay. Banco de Imagens. [Acesso em 18 de Agosto de 2021] 1280 x 938 pixels. Disponível em: https://pixabay.com/de/vectors/baden-sport-wasser-schwimmbad-2027088/

44. Como de levantar. [Imagem da Internet] "A cara da mãe" Blogspot [Acesso em 18 de Agosto de 2021] 226 x 130 pixels. Disponível em: http://acaradamae.blogspot.com/2011/08/forma-correta-para-acordar.html

Referências Imagens

45. Sentar e levantar da cadeira. [Imagem da Internet] Revista Pilates [Acesso em 18 de Agosto de 2021] 654 x 506 pixels. Disponível em: https://revistapilates.com.br/pilates-na-funcionalidade-do-joelho/

46. Elevação de calcanhar. [Imagem da Internet] “Roberto Ferraz” Blogspot [Acesso em 18 de Agosto de 2021] 520 x 600 pixels. Disponível em: http://blog.robertaferraz.com.br/bem-estar/treino-da-semana-membros-inferiores/

47. Marcha estacionária. [Imagem da Internet] “Actividad Fsica y Ejercicio” Blogspot [Acesso em 18 de Agosto de 2021] 520 x 600 pixels. Disponível em: http://actividadfisicayejercicioumb17.blogspot.com/2017/09/condicion-cardiorespiratoria.html

48. Ginásio Peso Esportes. [Imagem da Internet] Pixabay. Banco de Imagens. [Acesso em 18 de Agosto de 2021] 1280 x 1280 pixels. Disponível em: https://pixabay.com/pl/vectors/si%C5%82ownia-ci%C4%99%C5%BCary-sporty-148632/

Referências Imagens

49. Comprimido Capsúla Remédio. [Imagem da Internet] Pixabay. Banco de Imagens. [Acesso em 18 de Agosto de 2021] 1920 x 1152 pixels. Disponível em:https://pixabay.com/pt/illustrations/comprimido-c%c3%a1psula-rem%c3%a9dio-m%c3%a9dico-1884775/

50. Rede Companhia Social. [Imagem da Internet] Pixabay. Banco de Imagens. [Acesso em 18 de Agosto de 2021] 1920 x 1920 pixels. Disponível em:https://pixabay.com/pt/illustrations/rede-companhia-social-1020016/

51. Sangue Células Vermelho. [Imagem da Internet] Pixabay. Banco de Imagens. [Acesso em 18 de Agosto de 2021] 1920 x 960 pixels. Disponível em:https://pixabay.com/pt/illustrations/sangue-c%c3%a9lulas-vermelho-m%c3%a9dico-1813410/

52. A vitamina B.[Imagem da Internet] Pixabay. Banco de Imagens. [Acesso em 18 de Agosto de 2021] 1920 x 1080 pixels https://pixabay.com/pt/photos/a-vitamina-b-tablet-sucesso-copo-4308676/

Referências Imagens

53. Rim Renal Urinário. [Imagem da Internet] Pixabay. Banco de Imagens. [Acesso em 18 de Agosto de 2021] 1756 x 1920 pixels https://pixabay.com/pt/photos/a-vitamina-b-tablet-sucesso-copo-4308676/

54. Carne Comida Alimento. Imagem da Internet] Pixabay. Banco de Imagens. [Acesso em 18 de Agosto de 2021] 1920 x 986 pixels https://pixabay.com/pt/illustrations/carne-comida-alimento-alimenta%C3%A7%C3%A3o-2031564/

55. Saleiro Picante Pimenta. [Imagem da Internet] Pixabay. Banco de Imagens. [Acesso em 18 de Agosto de 2021] 735 x 1280 pixels https://pixabay.com/sk/vectors/so%c4%beni%c4%8dka-so%c4%be-trepa%c4%8dka-korenie-295473/

56. Ervas naturais farmacêuticas. [Imagem da Internet] Pixabay. Banco de Imagens. [Acesso em 18 de Agosto de 2021] 1440 x 1920 pixels https://pixabay.com/sk/photos/bylinky-prirodzen%c3%a9-farmaceutick%c3%bd-906140/

Referências Imagens

57. Colorau Salada Laranja. [Imagem da Internet] Pixabay. Banco de Imagens. [Acesso em 18 de Agosto de 2021] 1920 x 1090 pixels https://pixabay.com/pt/photos/colorau-salada-laranja-3212148/

58. Orgânicos. [Imagem da Internet] Secretária de Agricultura de São Paulo. Canal Rural [Acesso em 18 de Agosto de 2021] 768 x 507 pixels https://www.canalrural.com.br/tag/secretaria-de-agricultura-de-sao-paulo/

59. Copo de água. [Imagem da Internet] Pixabay. Banco de Imagens. [Acesso em 18 de Agosto de 2021] 1920 x 1090 pixel. Disponível em: https://pixabay.com/pt/photos/copo-de-%c3%a1gua-%c3%a1gua-copo-sede-4087606/

60. Ícones Telefone Arredondar. [Imagem da Internet] Pixabay. Banco de Imagens. [Acesso em 18 de Agosto de 2021] 1280 x 1280 pixel. Disponível em: https://pixabay.com/pt/vectors/%c3%adcones-telefone-arredondar-conectar-1831923/
