# Supplementary material for: Development and validation of a multidisciplinary educational booklet for kidney transplant recipients
Source: Rev Bras Enferm. 2025 Mar 14;78(1):e20240103. doi: 10.1590/0034-7167-2024-0103 (PMC11913046; doi:10.1590/0034-7167-2024-0103)
Supplement: Translation

# Translation: Construção e validação de uma cartilha educativa multiprofissional para transplantados renais

**DOI:** 10.1590/0034-7167-2024-0103pt

**Authors:** Juliane Custodio de Andrade, Andressa Santos Ferreira Brito, Kelly Aparecida Vieria dos Santos, Ariane Polidoro Dini, Angélica Gonçalves Silva Belasco, Renata Cristina Gasparino

**Affiliations:** I Universidade Estadual de Campinas. Campinas, São Paulo, Brasil; II Hospital Irmãos Penteado. Campinas, São Paulo, Brasil; III Universidade Federal de São Paulo. São Paulo, São Paulo, Brasil

**Keywords:** Estudos de Validação, Educação em Saúde, Tecnologia Biomédica, Segurança do Paciente, Transplante de Rim

## Abstract

**Objetivos:**

construir e validar o conteúdo de uma cartilha multiprofissional para pacientes submetidos a transplante renal.

**Métodos:**

estudo metodológico dividido em cinco etapas: 1) revisão bibliográfica; 2) organização dos domínios e inclusão de figuras; 3) validação do conteúdo; 4) formatação; e 5) avaliação do material pelo público-alvo. A validade de conteúdo foi analisada pelo Índice de Validade de Conteúdo (IVC) e Kappa modificado. Para análise da relevância, foi calculada a Razão de Validade de Conteúdo (RVC).

**Resultados:**

a cartilha foi construída e validada após quatro rodadas de avaliação, com média de IVC = 0,98, Kappa = 0,97 e RVC = 0,77. A avaliação do público-alvo alcançou 99,7% de concordância.

**Conclusões:**

a cartilha demonstrou evidências de validade de conteúdo, sendo clara, interessante e contendo informações necessárias, podendo ser utilizada por profissionais e pelo público-alvo.

## INTRODUÇÃO

O primeiro transplante renal (TX) ocorreu em Boston, em 1954^(1)^. No Brasil, a realização de TX renal teve início em 1964, e desde então, o número de procedimentos vem crescendo^(2)^. Diversas complicações podem ocorrer após o TX, especialmente a rejeição do órgão e o desenvolvimento de infecções, que predominam no primeiro ano após o procedimento^(3)^, por ser o período de maior utilização de imunossupressores^(4)^.

Para minimizar e até impedir a ocorrência desses eventos, é necessário investir, primeiramente, na educação em saúde do paciente, com o objetivo de estimulá-lo a implementar mudanças comportamentais^(5)^, direcionadas aos fatores de risco aos quais está submetido^(6)^.

O processo de educação em saúde vem apresentando grande evolução com o avanço científico e tecnológico e, consequentemente, o surgimento de diversas tecnologias educacionais (TE)^(7-9)^. O desenvolvimento de TE não deve ser limitado a materiais e equipamentos, mas deve possuir embasamento teórico, ser criativo, inovador, atrativo e acessível, de forma a potencializar o alcance dos objetivos, de acordo com a necessidade do público-alvo^(10)^.

Para a construção de materiais educativos, é necessária uma teoria consolidada. Como parte desse processo, é essencial a utilização de evidências científicas^(11)^, bem como a validação do material por especialistas na área, para uma análise minuciosa de todo o conteúdo^(12)^. Posteriormente, é imprescindível que ocorra a avaliação do material pelo público ao qual ele se destina^(13)^.

Ressalta-se a escassez de materiais educativos multiprofissionais na área de TX renal, e estudos de construção e validação desses materiais possuem grande potencial de impacto do ponto de vista técnico-científico, social e de inovação. Por isso, acredita-se que a construção de uma cartilha, considerada uma TE, será útil tanto para profissionais quanto para pacientes. Do ponto de vista dos profissionais, a cartilha poderá oferecer suporte, de forma padronizada, às orientações sobre os principais cuidados que os pacientes precisam implementar após o TX renal. Ao considerar os pacientes e cuidadores, essa ferramenta possibilitará o acesso rápido a informações construídas com base na literatura e validadas por especialistas, auxiliando no esclarecimento de dúvidas, prevenção de complicações e, consequentemente, contribuindo para a segurança do paciente.

## OBJETIVOS

Construir e validar o conteúdo de uma cartilha com orientações da equipe multiprofissional aos pacientes submetidos a TX renal.

## MÉTODOS

### Aspectos éticos

A pesquisa foi realizada de acordo com a Resolução 466/12 do Conselho Nacional de Saúde. O projeto foi aprovado pelo Comitê de Ética em Pesquisa (CEP) da instituição responsável pela coleta dos dados e pela universidade vinculada ao estudo. Todos os participantes assinaram o Termo de Consentimento Livre e Esclarecido (TCLE).

### Desenho, período e localização do estudo

Trata-se de um estudo metodológico, desenvolvido em cinco etapas: revisão bibliográfica; identificação e organização dos domínios e dos itens referentes às orientações que o paciente precisa receber das equipes de enfermagem, médica, farmácia, fisioterapia e nutrição, além da inserção de figuras lúdicas; validação de conteúdo; formatação da cartilha; e avaliação da cartilha pelo público-alvo^(13)^.

O estudo foi realizado no período de outubro de 2020 a fevereiro de 2022, em um hospital público universitário no interior do estado de São Paulo.

### Análise dos dados e estatística

Para avaliar a porcentagem de concordância entre os especialistas, foram calculados o Índice de Validade de Conteúdo (IVC)^(13)^ e o Kappa modificado^(14)^. Foram considerados satisfatórios os valores de IVC e Kappa modificado superiores a 0,90 e 0,74, respectivamente^(13,14)^. Para a avaliação da relevância, foi utilizada a Razão de Validade de Conteúdo (RVC), sendo que a razão mínima estipulada para o RVC foi igual ou superior a 0,78^(15)^. A avaliação da cartilha pelo público-alvo foi realizada por meio do cálculo de porcentagem de concordância.

### Amostra e critérios de inclusão e exclusão

Os critérios de inclusão para a amostra de especialistas foram: profissionais com especialização ou experiência clínica em nefrologia por no mínimo um ano. Foram excluídos os profissionais que não responderam ao formulário dentro do prazo estipulado. Para a amostra do público-alvo, foram incluídos 15 pacientes internados no hospital, que realizaram TX renal há no mínimo quatro dias e que estavam clinicamente estáveis no pós-operatório, e 15 pacientes que realizaram TX renal há um mês e estavam em acompanhamento ambulatorial. Em ambos os grupos, foi utilizado como critério de inclusão ter sido submetido ao TX renal pela primeira vez, ter mais de 18 anos e ser alfabetizado.

### Protocolo do estudo

Na primeira etapa, foi realizada uma revisão bibliográfica dos últimos cinco anos sobre os cuidados que o paciente submetido ao TX renal deve receber após a alta hospitalar. Para a coleta de dados, foi estabelecida a seguinte pergunta norteadora: *Quais são as principais orientações que os pacientes transplantados renais precisam receber para prevenir complicações?* Em seguida, foram definidos e utilizados os seguintes descritores: Estudos de validação, Educação em saúde, Tecnologia em saúde, Transplante renal, nas seguintes bases e portais: *National Library of Medicine and National Institute of Health* (PubMed), Literatura Latino-Americana e do Caribe em Ciências da Saúde (LILACS), *Medical Literature Analysis and Retrieval System Online* (MEDLINE), *Scientific Electronic Library Online* (Scielo), e *Cumulative Index to Nursing & Allied Health Literature* (CINAHL).

Após a seleção dos artigos relacionados, foi realizada a leitura dos resumos e a análise crítica dos estudos incluídos. Também foram consideradas informações presentes em protocolos disponibilizados pelo Ministério da Saúde (MS), Agência Nacional de Vigilância Sanitária (ANVISA) e Organização Mundial de Saúde (OMS). Nesse momento, foi estabelecida uma estrutura conceitual para a elaboração do conteúdo educativo multiprofissional da cartilha^(12,13)^.

Na segunda etapa, foram identificados os domínios e as orientações que os pacientes precisam receber das equipes de enfermagem, médica, farmácia, fisioterapia e nutrição. Posteriormente, foram selecionadas figuras ilustrativas gratuitas e sem direitos autorais, a fim de tornar a cartilha atrativa, acessível e de fácil compreensão.

Na terceira etapa, com o objetivo de avaliar a validade de conteúdo, foram convidados, por conveniência, dez profissionais da equipe multiprofissional (enfermeiros, farmacêuticos, fisioterapeutas, nutricionistas e médicos) envolvidos no atendimento de pacientes transplantados renais da instituição onde o estudo foi realizado. Os que aceitaram tiveram acesso a um formulário online, construído na plataforma Google Formulários^®^, cuja primeira tela continha o TCLE, seguido de um questionário para avaliar o perfil pessoal e profissional do participante.

Posteriormente, os participantes foram orientados a avaliar a clareza e representatividade do conteúdo. Para isso, em cada orientação descrita, foi inserida uma escala tipo Likert com cinco pontos: 0) Não consigo avaliar, pois o item encontra-se fora da minha área de atuação; 1) Item não claro e não representativo; 2) Item necessita de grande revisão para ser claro e representativo; 3) Item necessita de pequena revisão para ser claro e representativo; 4) Item claro e representativo.

Para avaliação da relevância, os especialistas utilizaram uma escala tipo Likert com quatro pontos: 0) Não consigo avaliar, pois o item encontra-se fora da minha área de atuação; 1) Item desnecessário; 2) Item útil, mas desnecessário; 3) Item essencial.

A avaliação do especialista que assinalou a opção de resposta zero não foi considerada para o cálculo do IVC, Kappa Modificado e RVC^(15)^.

Para os itens que não alcançaram os limites estabelecidos nos testes, as sugestões dos especialistas foram incorporadas para uma nova rodada de avaliação^(12,13)^. Após a etapa de validação da cartilha pelos especialistas, foi realizada a formatação da versão pré-final da cartilha para ser avaliada pelo público-alvo. A última etapa, denominada pré-teste, consistiu na avaliação da cartilha pelo público-alvo.

Os pacientes internados no hospital receberam a cartilha impressa e permaneceram com ela por 24 a 48 horas. Já os pacientes em atendimento ambulatorial permaneceram com a cartilha até o próximo retorno ambulatorial (dois a sete dias). Após esse período, foi solicitado aos pacientes o preenchimento de uma ficha de caracterização contendo dados pessoais e clínicos e um questionário com perguntas relacionadas à avaliação da organização/aparência, estilo e compreensão da escrita, além da motivação proporcionada pela cartilha^(16)^.

## RESULTADOS

Na primeira etapa do estudo, a revisão bibliográfica resultou em 25 artigos, duas teses e três protocolos, que foram analisados criticamente. A partir dessa revisão, na segunda etapa, 128 informações foram elaboradas e distribuídas conceitualmente em cinco domínios: 1) Orientações e cuidados gerais após o TX; 2) Recomendações sobre vacinação; 3) Recomendações sobre atividade física; 4) Uso de medicações; 5) Recomendações nutricionais, além de telefones úteis e referências.

Na terceira etapa, para o processo de validação de conteúdo, dos dez profissionais convidados, oito aceitaram participar, sendo seis (75,00%) do sexo feminino e três (37,50%) com experiência em estudos de validação. A média de idade foi de 35,38 anos (±6,63), e a média do tempo de atuação em nefrologia foi de 5,29 anos (±5,52). Na Tabela 1, são apresentadas as demais variáveis de caracterização da amostra de especialistas.

**Tabela 1:** Caracterização dos especialistas (n=8), Campinas, São Paulo, Brasil, 2021

Variável	n	%
Formação		
Enfermeiro	2	25,00
Farmacêutico	2	25,00
Fisioterapeuta	2	25,00
Nutricionista	1	12,50
Médico	1	12,50
Nível de formação		
Bacharelado	1	12,50
Pós-graduação lato sensu em nefrologia	2	25,00
Pós-graduação stricto sensu	5	62,50
Tempo que atua ou atuou na área de nefrologia		
<1 ano	1	12,50
2 a 5 anos	4	50,00
6 a 10 anos	2	25,00
>11 anos	1	12,50
Local que trabalha atualmente		
Outras Enfermarias	3	37,50
Enfermaria de Nefrologia	2	25,00
Ambulatório de Nefrologia	1	12,50
Outro	1	25,00
Cargo que ocupa atualmente		
Assistencial	2	25,00
Gerencial	2	25,00
Ensino/Educação	2	25,00
Assistencial/ Ensino/Educação	2	25,00

A cartilha foi enviada para os especialistas e após avaliação, foram obtidos os resultados referentes à clareza, representatividade e relevância de cada item (Tabela 2).

**Tabela 2:** Avaliação da clareza, representatividade e relevância dos itens na primeira rodada de avaliação pelos especialistas (n=8), Campinas, São Paulo, Brasil, 2021

Itens	IVC^*^	KAPPA	RVC¨	Conduta	Itens	IVC^*^	KAPPA	RVC¨	Conduta
**1**	1,00	1,00	1,00	Mantido	**65**	0,83	0,82	0,33	Excluído
**2**	1,00	1,00	0,75	Mantido	**66**	1,00	1,00	0,67	Mantido
**3**	1,00	1,00	1,00	Mantido	**67**	0,83	0,82	0,33	Manter/excluir
**4**	1,00	1,00	0,75	Mantido	**68**	0,83	0,82	0,33	Excluído
**5**	1,00	1,00	0,67	Mantido	**69**	1,00	1,00	1,00	Mantido
**6**	1,00	1,00	1,00	Mantido	**70**	1,00	1,00	0,67	Excluído
**7**	1,00	1,00	0,75	Mantido	**71**	1,00	1,00	0,67	Excluído
**8**	1,00	1,00	1,00	Mantido	**72**	0,83	0,82	0,33	Excluído
**9**	0,86	0,85	0,75	Modificado	**73**	1,00	1,00	0,67	Mantido
**10**	1,00	1,00	1,00	Mantido	**74**	1,00	1,00	0,43	Excluído
**11**	1,00	1,00	1,00	Mantido	**75**	1,00	1,00	0,43	Excluído
**12**	0,88	0,87	1,00	Modificado	**76**	1,00	1,00	0,43	Mantido
**13**	1,00	1,00	1,00	Mantido	**77**	0,86	0,85	0,71	Excluído
**14**	1,00	1,00	1,00	Mantido	**78**	0,86	0,85	0,71	Excluído
**15**	0,88	0,87	1,00	Modificado	**79**	0,86	0,85	0,71	Excluído
**16**	1,00	1,00	1,00	Mantido	**80**	0,86	0,85	0,71	Excluído
**17**	1,00	1,00	0,50	Mantido	**81**	0,86	0,85	0,71	Excluído
**18**	1,00	1,00	1,00	Mantido	**82**	0,86	0,85	0,71	Excluído
**19**	0,88	0,87	0,25	Excluído	**83**	0,86	0,85	0,71	Excluído
**20**	1,00	1,00	1,00	Mantido	**84**	0,86	0,85	0,71	Excluído
**21**	1,00	1,00	1,00	Mantido	**85**	0,86	0,85	0,71	Excluído
**22**	1,00	1,00	1,00	Mantido	**86**	0,86	0,85	0,71	Excluído
**23**	1,00	1,00	0,50	Mantido	**87**	0,86	0,85	0,71	Excluído
**24**	1,00	1,00	0,50	Mantido	**88**	0,86	0,85	0,71	Excluído
**25**	1,00	1,00	1,00	Mantido	**89**	0,86	0,85	0,71	Excluído
**26**	1,00	1,00	1,00	Mantido	**90**	0,86	0,85	0,71	Excluído
**27**	1,00	1,00	1,00	Mantido	**91**	0,86	0,85	0,71	Excluído
**28**	0,88	0,87	1,00	Modificado	**92**	0,86	0,85	0,71	Excluído
**29**	0,88	0,87	1,00	Modificado	**93**	0,86	0,85	0,71	Excluído
**30**	0,83	0,82	0,43	Excluído	**94**	1,00	1,00	1,00	Mantido
**32**	1,00	1,00	0,71	Manter/excluir	**95**	1,00	1,00	1,00	Mantido
**33**	0,83	0,82	0,43	Excluído	**96**	1,00	1,00	1,00	Mantido
**34**	1,00	1,00	0,43	Excluído	**97**	1,00	1,00	1,00	Mantido
**35**	0,83	0,82	0,43	Excluído	**98**	1,00	1,00	0,71	Mantido
**36**	0,83	0,82	0,43	Excluído	**99**	1,00	1,00	0,43	Mantido
**37**	0,83	0,82	0,71	Excluído	**100**	1,00	1,00	1,00	Mantido
**38**	1,00	1,00	0,43	Excluído	**101**	1,00	1,00	1,00	Mantido
**39**	0,83	0,82	0,43	Excluído	**102**	1,00	1,00	1,00	Mantido
**40**	0,83	0,82	0,43	Excluído	**103**	1,00	1,00	0,75	Mantido
**41**	1,00	1,00	1,00	Mantido	**104**	0,80	0,76	1,00	Modificado
**42**	1,00	1,00	1,00	Mantido	**105**	0,80	0,76	0,60	Manter/excluir
**43**	1,00	1,00	1,00	Mantido	**106**	0,80	0,76	1,00	Mantido
**44**	1,00	1,00	0,75	Mantido	**107**	0,80	0,76	0,60	Excluído
**45**	1,00	1,00	0,50	Mantido	**108**	1,00	1,00	0,60	Mantido
**46**	1,00	1,00	0,75	Mantido	**109**	0,80	0,76	1,00	Modificado
**47**	1,00	1,00	1,00	Mantido	**110**	1,00	1,00	1,00	Mantido
**48**	1,00	1,00	1,00	Mantido	**111**	0,75	0,67	-0,50	Excluído
**49**	1,00	1,00	1,00	Mantido	**112**	0,60	0,42	-0,20	Excluído
**50**	1,00	1,00	1,00	Mantido	**113**	0,60	0,42	-0,50	Excluído
**51**	1,00	1,00	0,67	Mantido	**114**	0,80	0,76	-0,50	Excluído
**52**	0,83	0,82	0,67	Modificado	**115**	1,00	1,00	1,00	Mantido
**53**	1,00	1,00	0,67	Mantido	**116**	0,80	0,76	-0,50	Excluído
**54**	1,00	1,00	0,67	Mantido	**117**	0,60	0,42	-1,00	Excluído
**55**	1,00	1,00	0,67	Mantido	**118**	0,80	0,76	0,20	Modificado
**56**	0,83	0,82	0,33	Modificado	**119**	1,00	1,00	1,00	Mantido
**57**	1,00	1,00	1,00	Mantido	**120**	0,80	0,76	0,60	Manter/excluir
**58**	1,00	1,00	1,00	Mantido	**121**	0,80	0,76	0,60	Excluído
**59**	1,00	1,00	0,67	Mantido	**122**	0,83	0,82	0,67	Excluído
**60**	1,00	1,00	0,67	Mantido	**123**	0,83	0,82	0,67	Excluído
**61**	0,83	0,82	0,33	Modificado	**124**	0,83	0,82	0,67	Excluído
**62**	1,00	1,00	0,67	Excluído	**125**	0,83	0,82	0,67	Modificado
**63**	1,00	1,00	0,67	Mantido	**126**	0,83	0,82	1,00	Manter/excluir
**64**	0,83	0,82	0,33	Modificado	**127**	1,00	1,00	1,00	Mantido
					**128**	1,00	1,00	1,00	Mantido

* *IVC - Índice de validade de conteúdo; ¨RVC - Razão de Validade de Conteúdo.*

Após a primeira avaliação, 38 itens alcançaram valores de 1,00 no IVC, Kappa Modificado e RVC, sendo mantidos sem qualquer modificação. O item 106 foi mantido, mesmo com valores de IVC e Kappa Modificado abaixo da referência e RVC de 1,00, pois não recebeu sugestões de alteração e foi considerado relevante por todos os especialistas. Outros 24 itens, apesar de terem apresentado uma RVC inferior a 0,78, não receberam a nota 1 (item desnecessário) por nenhum dos especialistas na avaliação da relevância. Como também não houve sugestões de modificação e os itens alcançaram IVC e Kappa Modificado de 1,00, foram mantidos sem alterações.

Entre o total de itens, sete apresentaram IVC e Kappa Modificado de 1,00, ou seja, estavam claros e representavam o domínio, mas valores de RVC insatisfatórios, e foram excluídos pelo fato de terem recebido a nota 1 (item desnecessário) por, pelo menos, um especialista da área. Outros 39 itens foram excluídos por apresentarem valores de IVC, Kappa Modificado ou RVC insatisfatórios, sem sugestões de alteração, e por terem recebido a nota 1 (item desnecessário) por, pelo menos, um especialista da área correspondente.

Cinco itens apresentaram valores de IVC, Kappa Modificado ou RVC insatisfatórios, porém não receberam nenhuma nota 1 (item desnecessário) nem sugestões de alteração. Por isso, esses itens retornaram para uma segunda rodada de avaliação, em que os especialistas foram questionados se o item deveria ser mantido ou excluído.

Para outros 13 itens, que alcançaram valores de IVC inferiores ao recomendado, mas receberam sugestões de modificação, optou-se por alterá-los e enviá-los para nova avaliação. Além dos 128 itens iniciais, os especialistas sugeriram a inclusão de mais 25 itens. Esses itens, somados aos 18 que apresentaram resultados insatisfatórios na primeira rodada, foram encaminhados para uma segunda rodada de avaliação.

Nesta segunda rodada, participaram sete especialistas, visto que um fisioterapeuta não respondeu ao questionário dentro do prazo estabelecido. Nessa etapa, três itens foram excluídos e outros três não alcançaram valores satisfatórios de IVC e Kappa Modificado, mas receberam sugestões de alteração.

No que se refere à avaliação da relevância, outros 13 itens obtiveram RVC inferior ao recomendado. No entanto, nenhum recebeu a nota 1 (item desnecessário), tampouco sugestões de alteração, e, por isso, foram mantidos como estavam, considerando que o IVC e o Kappa Modificado alcançaram valores máximos.

Na terceira rodada, participaram os mesmos especialistas da segunda, mas, ainda assim, não foi possível obter um consenso. Dessa forma, foi agendada uma reunião online com os membros do comitê. Participaram da quarta rodada apenas três especialistas (médico, enfermeira e nutricionista), por serem os especialistas da temática referida nos respectivos itens. Durante a reunião, os itens foram discutidos, modificados e, com o consenso de todos, mantidos com as alterações propostas.

Ao final desta etapa, a média global da avaliação de clareza e representatividade foi de IVC = 0,98 e Kappa Modificado = 0,97. Com relação à relevância dos itens que foram mantidos, a RVC foi de 0,77.

Após essa etapa, obteve-se a versão pré-final da cartilha de orientação multiprofissional para pacientes transplantados renais, constituída por 111 itens. Após a formatação, organização e ajustes realizados, 30 pacientes submetidos ao TX renal avaliaram a cartilha educativa, e 99,7% concordaram com a organização, o estilo da escrita, a aparência e a motivação proporcionada pela leitura da cartilha.

## DISCUSSÃO

O presente estudo construiu e validou o conteúdo de uma cartilha que auxiliará a equipe multiprofissional na orientação de pacientes submetidos a TX renal, facilitando o rápido e fácil acesso às informações necessárias para os pacientes e seus cuidadores no período pós-TX renal.

O fortalecimento da educação em saúde no cuidado pós-TX renal é considerado um fator essencial para o sucesso do procedimento, pois contribui para a diminuição de complicações e readmissões hospitalares^(17)^. Para que a transmissão de conhecimento ocorra de maneira efetiva, o uso de TE vem sendo cada vez mais explorado. A literatura apresenta a utilização de vídeos, mensagens de texto, documentos impressos^(9)^, aplicativos, websites e cartilhas^(10)^. Ressalta-se que, apesar do avanço da tecnologia, a disponibilização de cartilhas impressas ainda se faz necessária, especialmente para pacientes com acesso limitado a dispositivos móveis e internet.

Destaca-se que a cartilha, pela sua viabilidade de acesso a diferentes públicos, tem se mostrado um excelente instrumento de trabalho para os profissionais de saúde no processo educacional. Esses materiais vêm ganhando espaço, pois fornecem informações dentro de diversos contextos na área da saúde^(18-21)^.

A versão inicial da cartilha foi desenvolvida com 128 itens, distribuídos em cinco domínios. A etapa de construção foi embasada em evidências científicas obtidas por meio de revisão bibliográfica^(10,21-23)^. Após a busca dos artigos, foi realizada a leitura e análise crítica do material, extraindo-se as informações mais relevantes que responderam ao objetivo do estudo.

A etapa de validação de conteúdo é essencial para permitir a análise crítica dos itens que irão compor a cartilha, feita por especialistas, a fim de garantir que o material contenha informações necessárias e atualizadas, relacionadas ao objetivo proposto^(24)^.

O processo de validação de conteúdo foi realizado por uma equipe multiprofissional (enfermeiros, farmacêuticos, fisioterapeutas, nutricionista e médico), abrangendo assuntos de diversas áreas do conhecimento. Esses profissionais, assim como em outros estudos, possuíam experiência na temática e tinham diferentes níveis de formação, sendo a maioria com pós-graduação stricto sensu^(22,25-27)^. A composição de um comitê multiprofissional e experiente tem sido cada vez mais utilizada^(20,26,27)^ na validação de conteúdo, agregando contribuições significativas e maior qualidade às temáticas abordadas.

Oito especialistas participaram do estudo, número que está em consonância com diversos estudos de validação de conteúdo, que utilizaram no mínimo cinco profissionais^(20-22,26,27)^. Embora a literatura seja diversificada em relação à recomendação do número de especialistas^(25,28)^, o presente estudo seguiu o preconizado por autores nacionais^(13)^.

No presente estudo, foram necessárias quatro rodadas de avaliação. Ressalta-se que diversas pesquisas chegaram a um consenso entre os especialistas com apenas uma rodada de avaliação^(25-27,29)^. A realização de quatro rodadas pode refletir a expertise na temática e o alto nível de formação e comprometimento dos especialistas com a construção do material. Outra justificativa possível para a realização de quatro rodadas foi o fato de o presente estudo distinguir-se dos demais por utilizar mais de um cálculo para avaliar a validade de conteúdo, visto que muitas pesquisas com alta porcentagem de concordância entre especialistas utilizaram apenas o cálculo do IVC^(20,22,25,29)^.

A utilização da RVC com escala de três pontos também tornou a avaliação mais criteriosa, o que corrobora com outro estudo que utilizou o mesmo cálculo^(23)^. Pesquisas que empregaram a escala de quatro pontos para a avaliação da RVC apresentaram uma maior porcentagem de concordância desde a primeira rodada^(30)^.

Ao final da etapa de validação de conteúdo, a versão pré-final da cartilha ficou composta por 111 itens, divididos em quatro domínios: 1) Orientações e cuidados gerais após o TX, com foco na prevenção e identificação de complicações; 2) Recomendações e contraindicações relacionadas às atividades físicas no período pós-TX; 3) Uso de medicações, com ênfase em imunossupressores; e 4) Recomendações nutricionais (alimentação saudável, cuidados no preparo dos alimentos e ingestão de água), além de telefones úteis e referências.

Esses domínios vão ao encontro das necessidades relatadas por pacientes em um estudo prévio, no qual as dificuldades apontadas estavam relacionadas à realização de tarefas diárias, rotinas de medicação, dieta e exercícios^(31)^.

Na última etapa do estudo, a avaliação da cartilha pelo público-alvo demonstrou um alto nível de concordância entre os participantes, permitindo afirmar que a cartilha desenvolvida está adequada para o público ao qual se destina. Os pacientes que participaram desta etapa avaliaram a cartilha quanto à organização, estilo da escrita, aparência e motivação para leitura, assim como em outros estudos^(18,31,32)^. Entretanto, destaca-se que outras pesquisas não realizaram essa etapa fundamental para avaliar a clareza, o interesse e a motivação que o material desperta no público-alvo^(20,21,23,28)^.

A inclusão de imagens relacionadas aos temas abordados tornou a cartilha mais atrativa, despertando o interesse do leitor. Para a cartilha desenvolvida no presente estudo, as imagens sem direitos autorais foram retiradas da internet, sendo consideradas de boa qualidade e eficazes em atrair a atenção dos leitores, o que difere de outras pesquisas que utilizaram um design específico para material educativo ou comunicação^(18,19,25)^.

A cartilha construída e validada encontra-se disponível^(33)^ para uso, e novos estudos poderão ser delineados com o objetivo de testar a eficácia do processo educacional com a utilização desse material, o empoderamento dos pacientes e o impacto na diminuição de complicações.

### Limitações do estudo

O comitê foi composto por especialistas do local do estudo e selecionado por conveniência, o que pode representar mais as especificidades da instituição na qual a pesquisa foi realizada. Entretanto, cabe salientar que a maioria dos temas abordados reflete os principais estudos sobre o cuidado pós-TX. Além disso, a ausência de um design específico na elaboração e/ou avaliação do material também pode ser considerada uma limitação no desenvolvimento da tecnologia.

### Contribuições para a área

Acredita-se que a utilização dessa TE na prática clínica e no ensino contribuirá para a padronização de informações baseadas em evidências, abrangendo os assuntos mais relevantes no cuidado pós-TX. Espera-se que a disponibilização desse material facilite o acesso às informações de maneira rápida, clara e objetiva, e que os pacientes se empoderem do seu autocuidado, garantindo sua própria segurança.

## CONCLUSÕES

A cartilha foi construída com base na literatura e demonstrou possuir evidências de validade de conteúdo, sendo clara, interessante e contendo informações necessárias ao público-alvo.

## Data Availability

https://doi.org/10.20396/ISBN9786587100326 PDF
